# Adolescent social instability stress alters social processes in male prairie voles

**DOI:** 10.3389/fnbeh.2026.1761549

**Published:** 2026-02-24

**Authors:** Lindsay L. Sailer, Amit Hanadari-Levy, Alexander G. Ophir

**Affiliations:** Department of Psychology, Cornell University, Ithaca, NY, United States

**Keywords:** anterior cingulate cortex (ACC), dopamine, lateral septum (LS), male prairie voles (*Microtus ochrogaster*), nucleus accumbens (NAc), oxytocin, social instability stress, vasopressin

## Abstract

Adolescence is a sensitive period for the maturation of neural circuits governing goal-directed social behaviors and stress regulation. Disruption of stable social relationships during adolescence can alter neuropeptide and dopaminergic systems that shape adult social behaviors. We investigated the behavioral and neurobiological consequences of adolescent social instability stress (SIS) in male prairie voles (*Microtus ochrogaster*), a species that forms selective social bonds between peers, mating partners, and parents and their offspring. During adolescence, SIS subjects experienced repeated reshuffling of cage mates to disrupt stable peer bonds, while control (CTL) subjects remained in fixed pairs. Home cage observations after and right before each reshuffling revealed that SIS subjects exhibited reduced affiliative contact and sustained social investigation compared to CTL subjects, despite no group differences in body weight throughout adolescence. Moreover, SIS and CTL groups did not differ in social zone duration or latency to approach a novel conspecific during the social approach test (SAT). Stress phenotypes were classified by assessing the duration of social zone occupancy during the SAT under baseline and stimulus-present conditions. Remarkably, all SIS subjects expressed a consistent stress resilient phenotype in contrast to CTL subjects whose responses were more variable, spanning both stress resilient and susceptible phenotypes. Gene receptor expression analyses revealed no group differences in oxytocin (*Oxtr*), arginine vasopressin (*Avpr1a*), and dopamine (*Drd1* and *Drd2*) gene expression within the lateral septum (LS), nucleus accumbens (NAc), or anterior cingulate cortex (ACC), brain regions important for modulating goal-directed social behaviors and stress responses. However, correlation analyses indicated distinct relationships between gene receptor expression and social behaviors across groups, including a negative association between LS- *Avpr1a* expression and the latency to approach a novel conspecific in only CTL subjects. Additionally, associations between ACC-*Drd2* expression and the latency to approach a stimulus were in opposing directions between groups. Correlation analyses solely between gene receptor expression revealed the loss of oxytocin-dopamine receptor coupling in the LS and ACC of SIS but not CTL subjects. Together, these findings suggest that adolescent SIS does not globally suppress social behavior but instead may reorganize social reward circuitry to promote behavioral flexibility and stress resilience.

## Introduction

The integrity of adult brain function and behavior is profoundly shaped by experiences during sensitive developmental periods. Adolescence, a transitional stage characterized by rapid social and neurobiological maturation, represents a critical window of vulnerability to environmental stressors (Andersen and Teicher, 2008; Tottenham and Galvan, 2016). Social adversity encountered during this period can profoundly alter neural circuitry, producing enduring effects on emotional regulation and social functioning. Such experiences are linked to long-lasting impairments in adult social behaviors, such as reduced affiliation and heightened aggression, as well an increased risk for mood disorders, including anxiety and depression.

The Social Instability Stress (SIS) paradigm, which is based in principles of neuroethology for its ecological relevance, has been used to effectively model the complexity of social stress and has been used to understand the effects of adverse social environments on brain and behavior (Mccormick et al., 2015; Burke et al., 2017; Koert et al., 2021). In this paradigm, repeated reorganization of cage-mates disrupts the formation and maintenance of stable social relationships, effectively modeling the instability of a dynamic social environment. In principle, the SIS paradigm maps onto the everchanging social dynamics of social turnover of group living animals, and SIS is considered ecologically relevant to humans because it mirrors experiences faced by children and adolescents exposed to residential instability, unstable family structures, or peer rejection (Zehrung et al., 2024; Fomby and Sennott, 2013). These forms of social stress disrupt critical social bonds, alter brain development, and can potentially lead to reduced social motivation.

The present study adapts this robust SIS paradigm to the prairie vole (*Microtus ochrogaster*), a socially monogamous rodent that exhibits strong and enduring sex-naïve peer bonds and mating-induced pair bonds (Getz et al., 1981; Getz et al., 1993; Lee and Beery, 2021). The prairie vole offers a valuable opportunity to investigate the neurobiology of social attachment, because its socially selective behaviors can more closely parallel human social relationships than those of traditional laboratory rodents. Social behaviors in prairie voles are orchestrated by the coordinated actions of oxytocin (OXT), arginine vasopressin (AVP), and dopamine (DA) signaling within limbic and cortical circuits associated with social bonding and stress regulation (Aragona and Wang, 2009; McGraw and Young, 2010). Specifically, oxytocin receptor (*Oxtr*) and arginine vasopressin receptor 1a (*Avpr1a*) gene expression and/or the protein receptors they encode in the lateral septum (LS), nucleus accumbens (NAc), and anterior cingulate cortex (ACC) modulate social recognition, pair bonding, and empathy-related behaviors (Lim and Young, 2006; Prounis and Ophir, 2020). As a central node of the Social Decision-Making Network (O’connell and Hofmann, 2012), the LS integrates social and emotional signals, and it contributes to social recognition, social behaviors, and anxiety regulation (Singewald et al., 2003; Sheehan et al., 2004). The NAc, a central component of the mesolimbic reward pathway, mediates motivational aspects of affiliative and partner-directed behaviors through dopaminergic signaling (Liu and Wang, 2003; Aragona et al., 2006). Within the NAc, the dopamine receptor subtypes D1R (*Drd1*) and D2R (*Drd2*) play opposing roles, where D2R activation facilitates pair bond formation and D1R activation is associated with the maintenance of selective affiliation and the exclusion of novel conspecifics (Aragona et al., 2006). The ACC contributes to the evaluation of social context, empathy-like processes, and adaptive responses to social stress (Allsop et al., 2018; Prounis and Ophir, 2020).

Extensive research focusing on the physiological and behavioral impacts of social instability stress with non-monogamous rodents has revealed profound effects on stress systems, emotional behaviors, and cognitive functions (Mccormick and Green, 2013; Koert et al., 2021; Mccormick et al., 2015). However, the longstanding effects of adolescent social instability stress on sub-adult social behaviors in a species capable of forming selective attachment to conspecifics remain poorly understood. This gap limits our understanding of how early-life social adversity influences the neural systems that govern experience-dependent social motivation, affiliative responses, and stress susceptibility. To bridge this gap, the present study examined the behavioral and neurobiological consequences of adolescent SIS in male prairie voles. We hypothesized that exposure to SIS during adolescence would disrupt affiliative and investigative interactions necessary for the formation of peer bonds and lead to persistent deficits in sub-adult social approach and enhance stress susceptible phenotypes. Furthermore, we predicted that these behavioral outcomes would be associated with dysregulation of *Oxtr, Avpr1a, Drd1*, and *Drd2* mRNA expression within the LS, NAc, and ACC.

## Materials and methods

### Animals

Prairie voles (*N* = 20) used in this experiment were F1–F2 generation descendants bred from wild-caught voles originally trapped in Champaign-Urbana, IL and raised by both parents. All animals were housed in transparent polycarbonate rodent cages (46.5 × 25 × 15.5 cm) lined with Sani-chip bedding (P. J. Murphy Forest Products, Montville, NJ, United States). All animals were given wooden chew blocks and cotton nesting material for environmental enrichment, and *ad libitum* access to food (Laboratory Rodent Diet 5001, LabDiet, St. Louis, MO, United States) and water. Sex was determined based on external genitalia. At weaning on postnatal day (PND) 21, male subjects were ear-tagged and loosely fitted with zip-ties around their necks for identification. From PND21 until the start of testing on PND31, male subjects were pair-housed with a male sibling. Handling was limited to weekly cage changing days. The experimental procedures followed the guidelines of the National Institutes of Health and were approved by the Cornell University Institutional Animal Care and Use Committee (protocol # 2013-0102).

### Social instability stress paradigm

**FIGURE 1:**
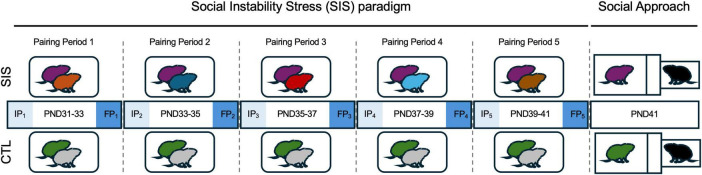
Experimental design. During the social instability stress (SIS) paradigm, subjects (purple) were re-paired with a novel conspecific (also serving as an SIS subject) every 2 days from PND31 to PND41 for a total of 5 Pairing Periods. CTL subjects (green) remained pair-housed with the same littermate throughout PND31-41 but were placed in a clean cage every 48 h. Social interactions were recorded in the first 5 min (Introductory Period 1–5, IP1–5) of subjects being placed in a new home cage at the beginning of each Pairing Period. The last 5 min of the 2-day cohabitations were recorded (Familiarity Period 1–5, FP1–5) for each Pairing Period. Change within each pairing period (ΔPPn) was calculated as the difference between FPn and IPn (FPn - IPn). Sociability of the SIS and CTL groups was assessed in the Social Approach Test on PND41 (also see Figure 2). Brains were extracted after subjects were sacrificed (within 10 min after the SAT).

At weaning, paired subject littermates were randomly assigned to either the social instability stress (SIS; 10 males) group or the control (CTL; 10 males) group. Subjects in the SIS group were paired with a different SIS individual every 2 days in a new cage over the course of 10 days, with each subject experiencing five different social encounters (i.e., “Pairing Periods”) from PND31-41 (Figure 1). To minimize the number of animals needed for this study, the 10 males used in the SIS condition were used to create each of the five unique pairings for each male. For each male, the series of pairing periods was unique to that male. Thus, both males in each SIS pair over the course of the study served as a focal subject; however, the series of pairings (i.e., social encounters) were unique to each subject (see statistical analyses below). CTL subjects were placed in a new cage with the same littermate every 2 days across all pairing periods, which controlled for any stress-induced effects by frequent cage changes.

**FIGURE 2:**
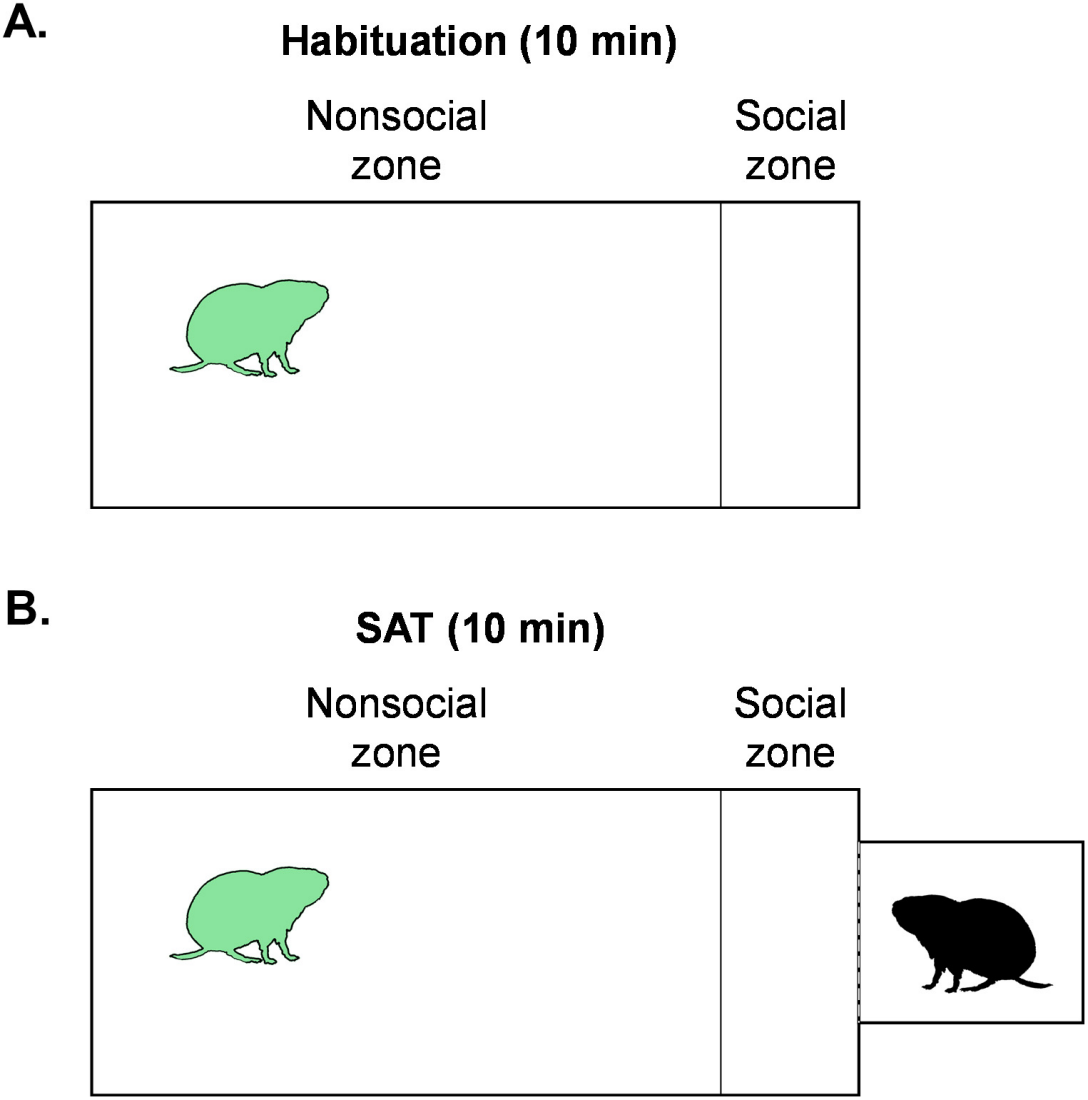
Experimental setup for the social approach test (SAT). **(A)** The subject (green vole) was placed in the main chamber for 10 min of habituation in the absence of a stimulus animal. **(B)** Subjects were briefly removed from the main chamber and a presentation chamber containing a stimulus animal (black vole) was attached to the main chamber. The subject was returned to the main chamber for another 10 min to assess social approach behaviors. The social zone was defined by the distance from the wall attached to the stimulus box in which an average prairie vole (3 cm length) could fit.

### Home cage observations during the SIS paradigm

Home cage observations between subjects in the same group were conducted to characterize spontaneous, naturalistic, and physical social behaviors. We recorded the first 5 min when subjects were introduced to either a novel conspecific (in the SIS condition) or the same littermate (in the CTL condition) during the five home cage pairing periods on PND31, 33, 35, 37, and 39 (Figure 1). These initial social encounters were intended to capture the introductory phase of the pairing period. We also recorded the last 5 min of the 2-day pairing period on PND33, 35, 37, 39, and 41 to capture the social encounters after familiarity was established. We refer to these two recording phases as the Introductory Period (IP) and the Familiarity Period (FP), respectively (Figure 1). For each pair, the total time engaged in social behaviors between conspecifics was scored because zip tie colors were blocked by fur during substantial portions of the recordings and the video resolution was not optimal making it difficult to discriminate between cagemates in video recordings. Home cage observation sessions were scored using BORIS (version 8.23) behavioral coding software (Friard and Gamba, 2016) for huddling, allo-grooming, social investigation (flank sniffing, anogenital sniffing, following, and nose-to-nose sniffing), and aggression (chasing and wrestling). The first familiarity period (FP_1_) between animals in CTL3 and CTL4 had to be excluded due to technical difficulties.

### Body weight measurement

The body weight of each subject was recorded on each cage-change day (PND31, 33, 35, 37, 39, and 41). The overall body weight gain was calculated (PND41 body weight—PND31 body weight) and used as a measure of physical development in response to social instability stress.

### Social approach test

All subjects underwent a Social Approach Test (SAT) after the FP_5_ on PND41 (Figures 1, 2). The SAT enabled us to evaluate social approach under conditions that preclude direct physical contact and therefore to isolate approach motivation independent of reciprocal social interaction or aggression and stress phenotypes (Sailer et al., 2022b; Golden et al., 2011). The social approach apparatus consisted of a main testing chamber (20 × 40 × 28 cm) with a doorway that can be blocked or serve as an attachment point for a presentation chamber (10.06 cm^3^) with a perforated plexiglass wall between the chambers. The perforated wall allows olfactory, visual, and auditory interactions between the subject and stimulus animal, while preventing physical contact. Subjects were habituated to the main testing chamber and testing room for 10 min; no presentation chamber was attached, and the doorway was blocked by a 10.06 cm^2^ plexiglass barrier (Figure 2A). At the same time, a novel sex-matched and age-matched stimulus animal was habituated in the presentation chamber. Subjects were briefly removed from the main testing chamber, the barrier was removed, and the presentation chamber containing the stimulus animal was attached. The SAT began when subjects were returned to the main testing chamber (Figure 2B). Interactions with the stimulus were recorded for 10 min. The section of the testing chamber closest to the stimulus animal, measured as one body length of a vole (within 3 cm of the stimulus chamber), was used to define the “social zone.” The rest of the testing chamber was considered the “non-social zone.”

The videos from the SAT were scored and analyzed using Noldus Observer XT13 video scoring software (Noldus, 1991) to measure time spent in the social and non-social zones, latency to approach a stimulus, Social Investigation (SI) ratio (time spent in the social zone with the stimulus present divided by the time spent in the social zone with the stimulus absent), frequency of visits into each zone, distance moved, and velocity. The SI ratio was used to define stress susceptible and stress resilient phenotypes (Sailer et al., 2022b; Golden et al., 2011). Subjects with SI ratios < 1.0 were defined as displaying stress susceptibility and subjects with SI ratios > 1.0 were defined as displaying stress resiliency. One SIS animal had to be excluded from the SI ratio analysis because it did not enter the social zone when the stimulus animal was absent and its SI ratio was undefined.

### Tissue processing

Subjects were euthanized following recommended ethical and regulatory guidelines: with CO_2_ asphyxiation (displaced between 30 and 70% of the chamber volume per minute) before rapid decapitation on PND41 immediately after the SAT. Brain tissue was immediately extracted and frozen on powdered dry ice before being stored at −80°C. Coronal sections (200 μm) were selected that anatomically matched Plates 14–34 for the lateral septum (LS), Plates 15–25 for the nucleus accumbens (NAc) and cingulate cortex (ACC) from Paxinos and Watson’s rat brain atlas (Paxinos and Watson, 2007). Tissue punches (1 mm diameter) were collected bilaterally from the LS, NAc, and ACC for each subject, and stored at −80°C until further processing for total RNA extraction using TRI-reagent according to the manufacturer’s protocol (Molecular Research Center) and as previously described (Wang et al., 2013; Sailer et al., 2019; Sailer et al., 2022b).

### Gene receptor expression analysis

Total RNA (200 ng) was reverse-transcribed with the LunaScriptTM RT SuperMix Kit (New England Biolabs, E2010) to examine the mRNA expression for *Oxtr*, *Avpr1a*, *Drd1*, and *Drd2* RT-qPCR in triplicates (see Supplementary Table S1 for primer sequences) for each subject. Primer specificity was verified by the melt curve analysis. For each primer pair, amplified cDNA was normalized to nicotinamide adenine dinucleotide dehydrogenase (*Nadh*) (Wang et al., 2013; Sailer et al., 2019; Sailer et al., 2022b). All data were included in the analyses unless statistically defined as an outlier (> 2 standard deviations from the mean).

### Statistical analyses

**FIGURE 3:**
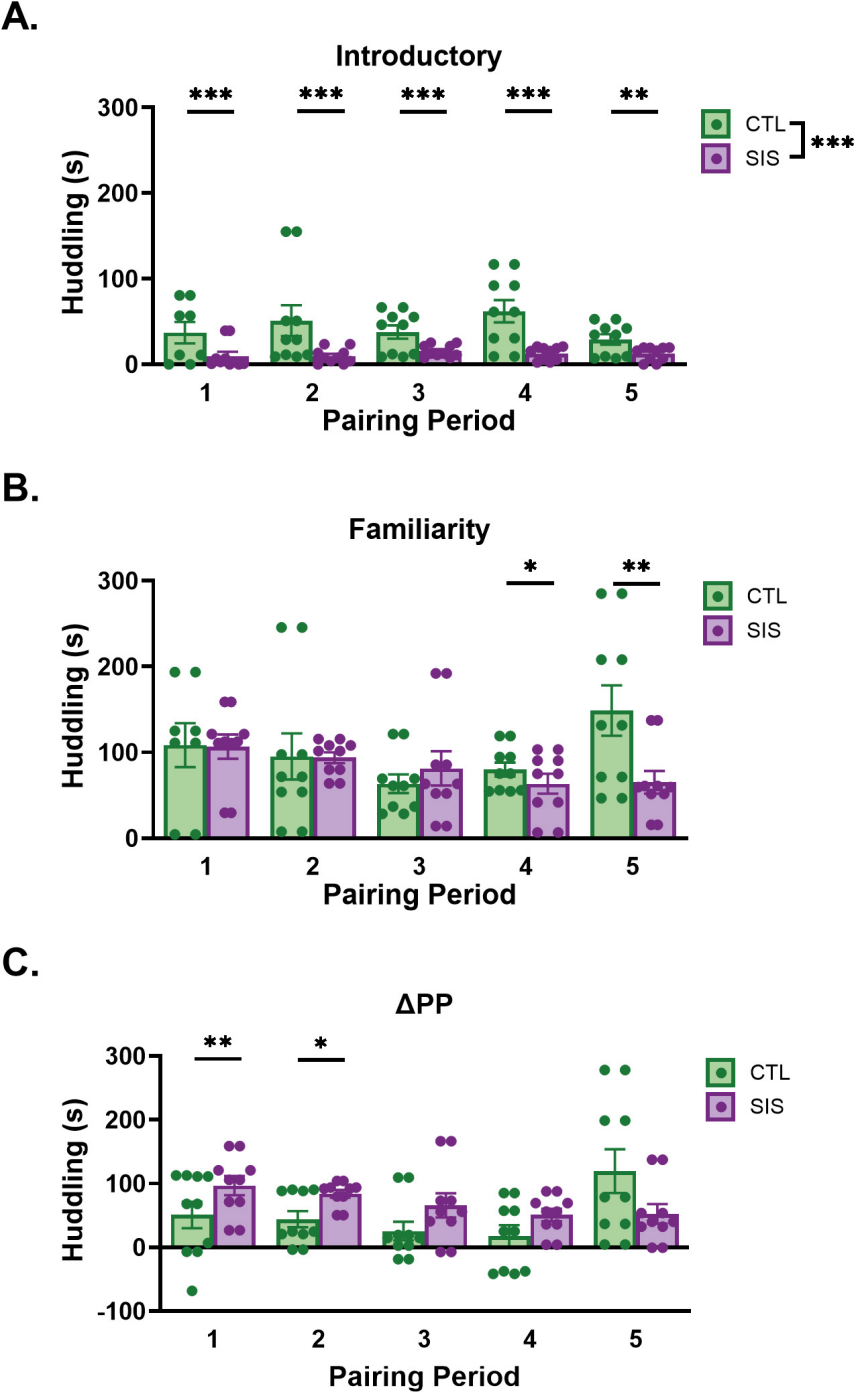
Duration of huddling during home cage observation. Introductory Periods 1–5 **(A)** occurred during postnatal days 31, 33, 35, 37, and 39, respectively. Familiarity Periods 1–5 **(B)** occurred during postnatal days 33, 35, 37, 39, and 41, respectively. **(C)** Change in huddling within Pairing Period (ΔPPn = FPn - IPn). Data are presented as mean ± SEM and dots represent individual data (*n* = 8–10/group); CTL group shown in green dots and bars; SIS group shown in purple dots and bars; *p < 0.05; ^**^p < 0.01; ^***^p < 0.001.

**FIGURE 4:**
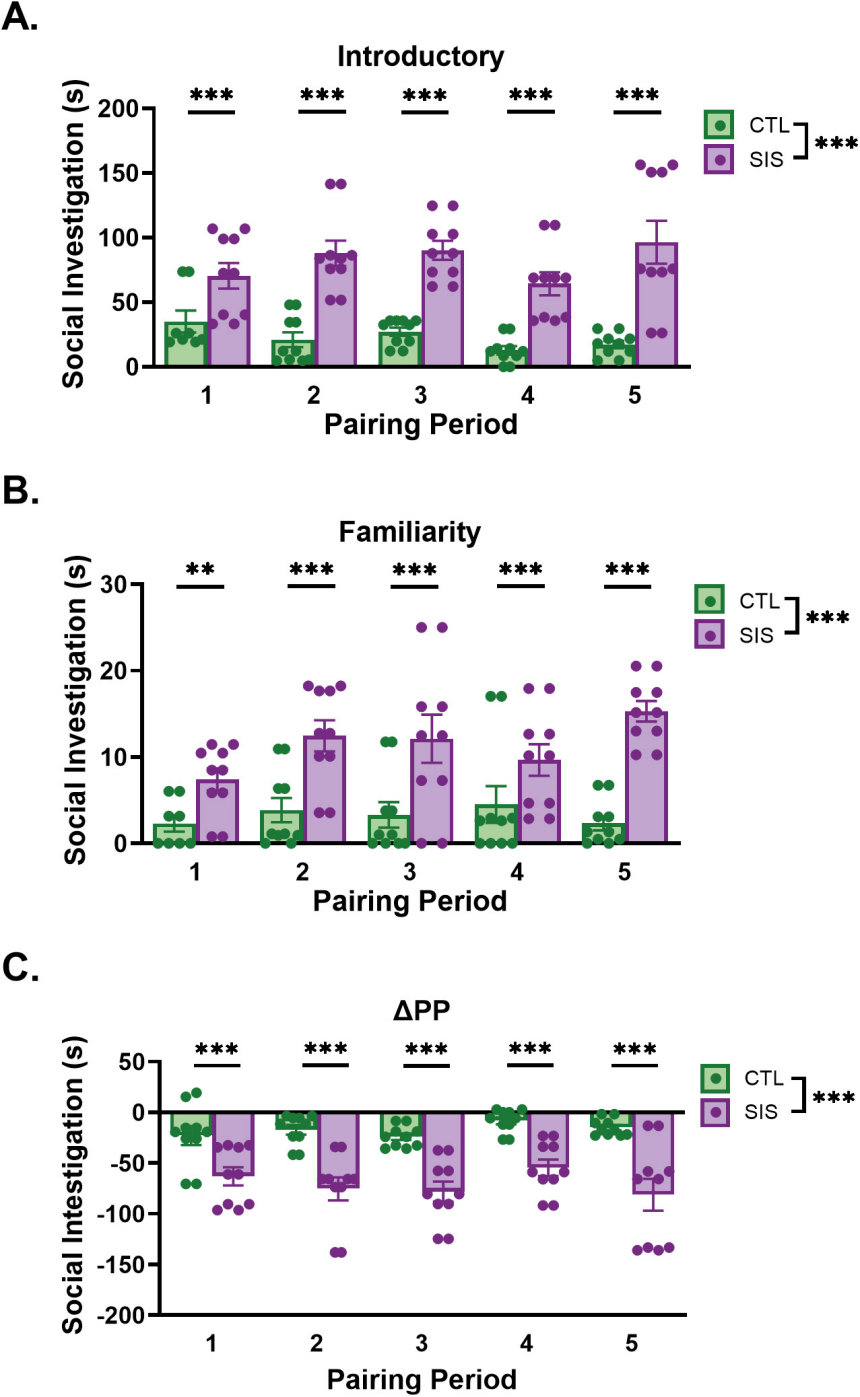
Duration of social investigation during home cage observation. Introductory Periods 1–5 **(A)** occurred during postnatal days 31, 33, 35, 37, and 39, respectively. Familiarity Periods 1–5 **(B)** occurred during postnatal days 33, 35, 37, 39, and 41, respectively. **(C)** Change in social investigation within Pairing Period (ΔPPn = FPn - IPn). Note that the y-axes differ between panels AC. Data are presented as mean ± SEM and dots represent individual data (*n* = 8–10/group); CTL group shown in green dots and bars; SIS group shown in purple dots and bars; ^**^p < 0.01; ^***^p < 0.001.

**FIGURE 5:**
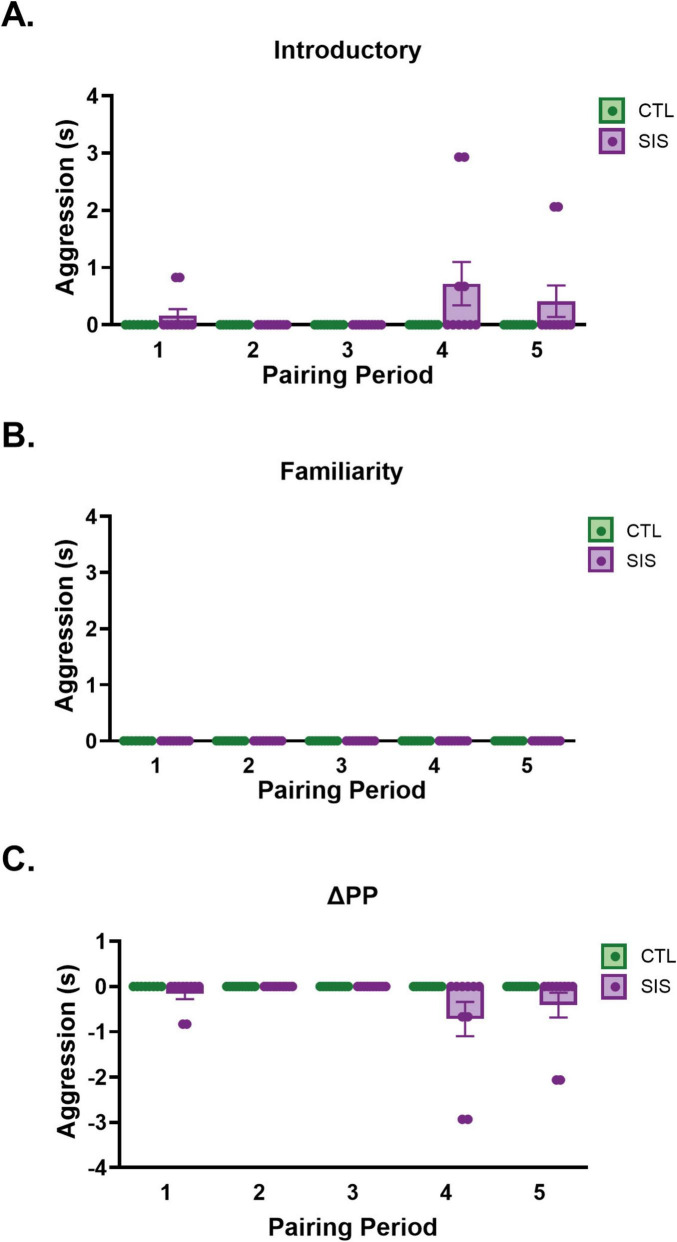
Duration of aggression during home cage observation. Introductory Periods 1–5 **(A)** occurred during postnatal days 31, 33, 35, 37, and 39, respectively. Familiarity Periods 1–5 **(B)** occurred during postnatal days 33, 35, 37, 39, and 41, respectively. **(C)** Change in aggression within Pairing Period (ΔPPn = FPn - IPn). Data are presented as mean ± SEM and dots represent individual data (*n* = 8–10/group); CTL group shown in green dots and bars; SIS group shown in purple dots and bars.

**FIGURE 6:**
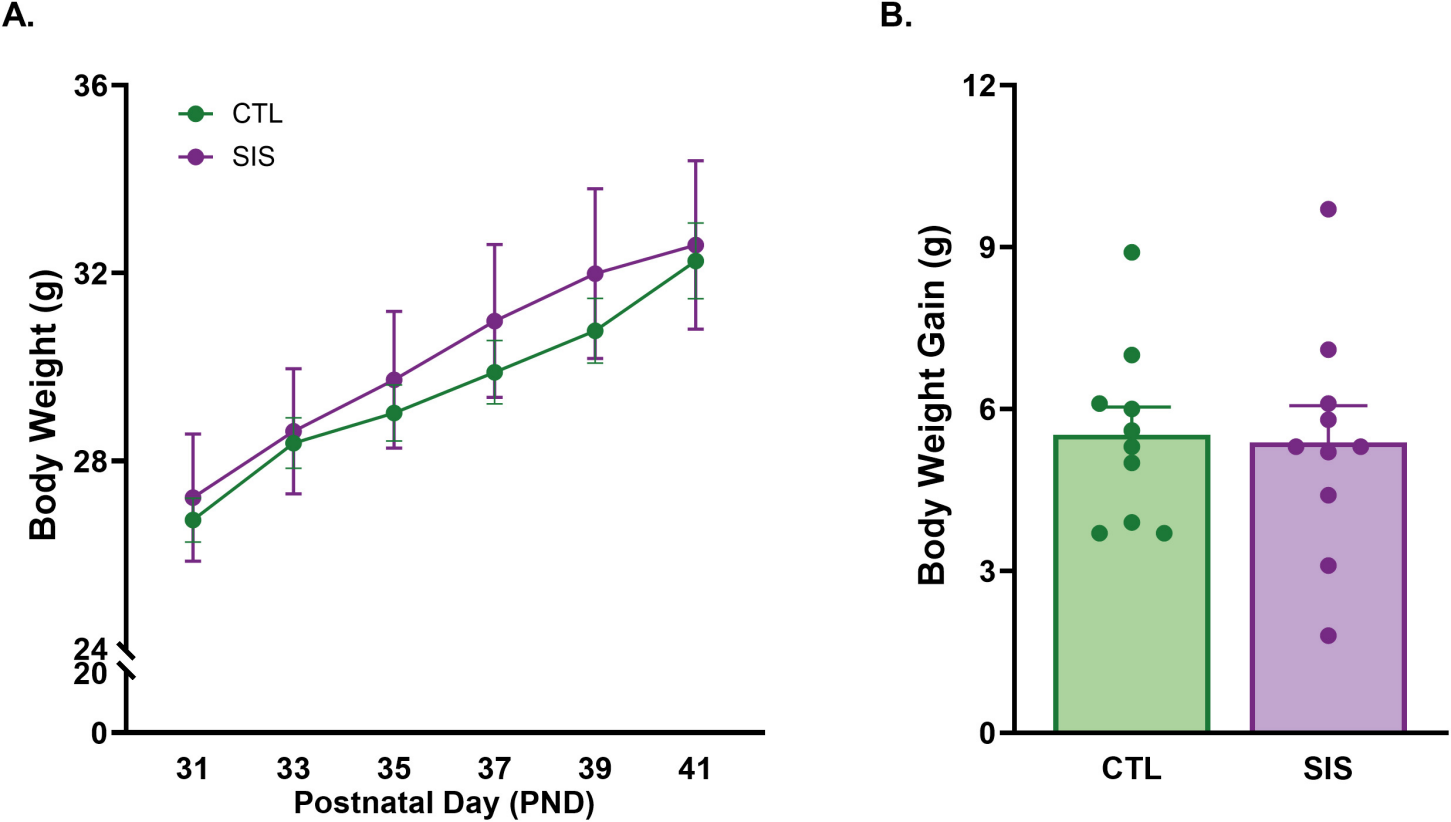
Social instability stress does not impact physical development during adolescence. **(A)** Body weight in grams for subjects at every cage switch day of the SIS paradigm. **(B)** Body weight gain from PND31 to PND41 (= PND41-PND31). Data are presented as mean ± SEM and dots represent individual data (*n* = 10/group); CTL group shown in green dots and bars; SIS group shown in purple dots and bars.

**FIGURE 7:**
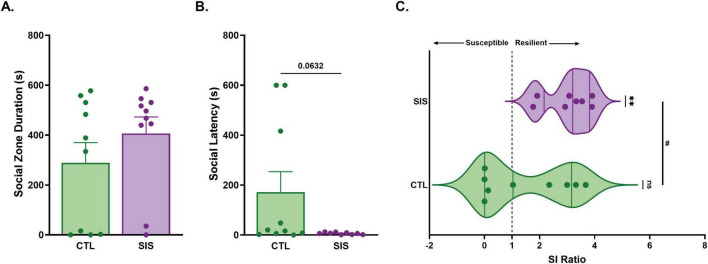
Social instability stress promotes stress resilience. **(A)** Subjects in the CTL and SIS groups spent similar durations in the social zone of the social approach test. **(B)** SIS subjects tended to display a shorter latency to approach a novel sex- and age-matched stimulus. **(C)** The SI ratios between CTL and SIS groups were significantly different and only the SI ratios of the SIS group significantly differed from the theoretical median of 1. Data are presented as mean ± SEM and dots represent individual data (*n* = 8–10/group); CTL group shown in green dots and bars; SIS group shown in purple dots and bars. Comparison between groups: ^#^*p* < 0.05; group theoretical median comparison to theoretical median: ^**^p < 0.01.

**FIGURE 8:**
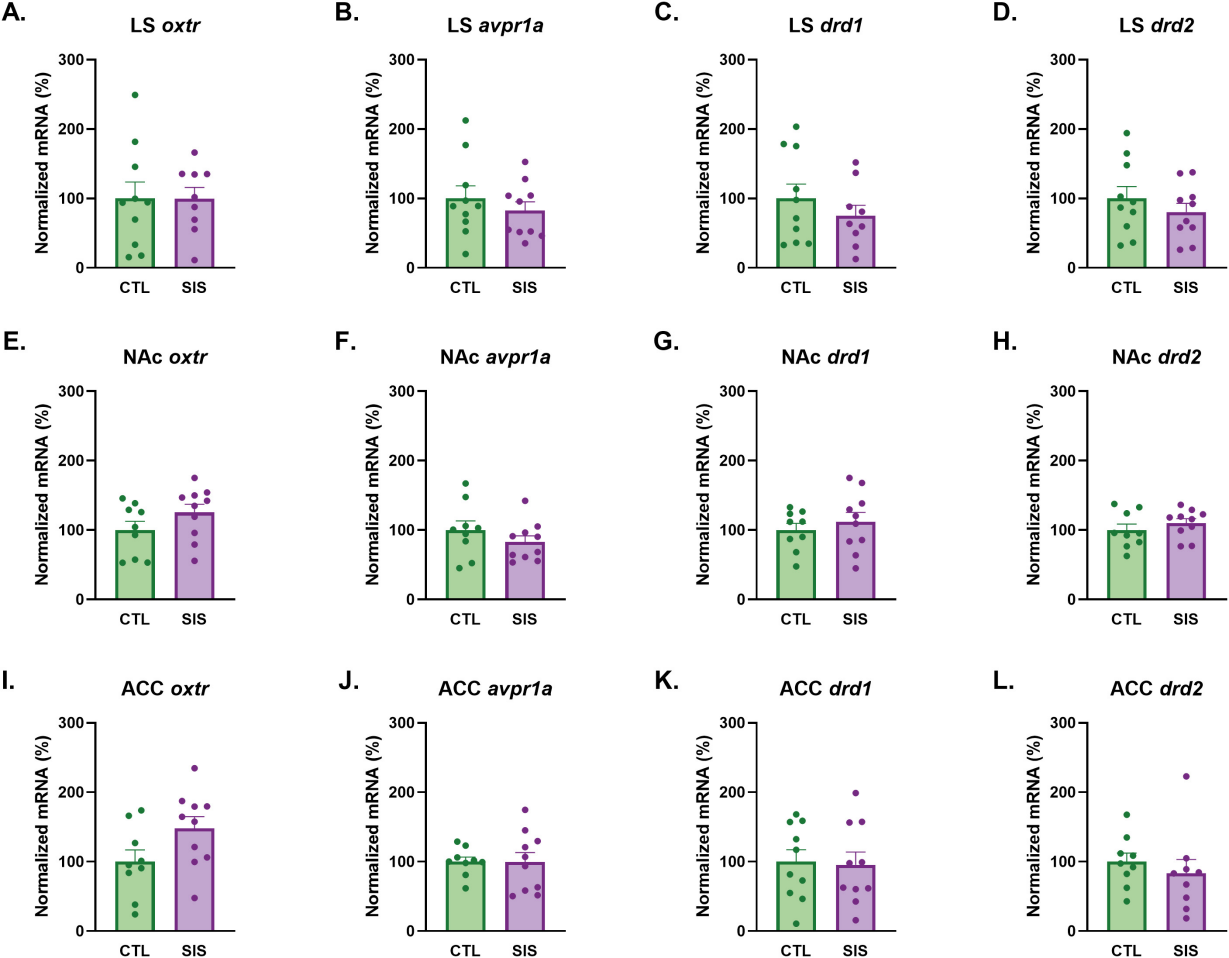
Effects of social instability stress on gene expression in the lateral septum (LS), nucleus accumbens (NAc), and anterior cingulate cortex (ACC). **(A–D)** LS mRNA expression of *Oxtr*, *Avpr1a*, *Drd1*, and *Drd2*. **(E–H)** NAc mRNA expression of *Oxtr*, *Avpr1a*, *Drd1*, and *Drd2*. **(I–L)** ACC mRNA expression of *Oxtr*, *Avpr1a*, *Drd1*, and *Drd2*. Data are presented as mean ± SEM and dots represent individual data (*n* = 9–10/group); CTL group shown in green dots and bars; SIS group shown in purple dots and bars.

**FIGURE 9:**
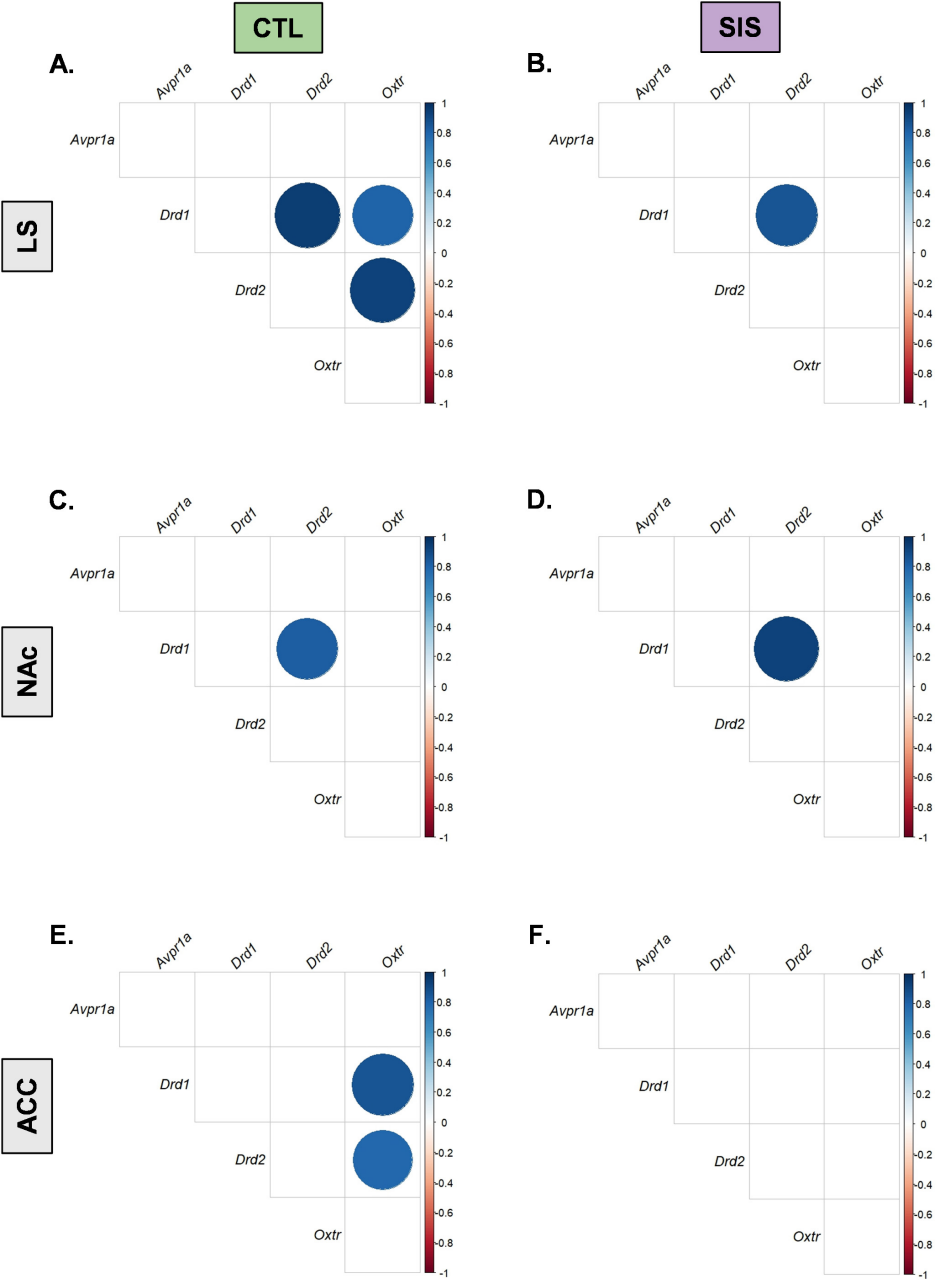
Correlograms separated by group and brain region. Pair-wise Pearson correlations with false discovery rate were calculated for mRNA expression by group in the LS **(A,B)**, NAc **(C,D)**, and ACC **(E,F)**. Positive correlations are displayed in blue and negative correlations in red color. Color intensity and the size of the circle are proportional to the correlation coefficients. In the right side of each correlogram, the legend color shows the correlation coefficients and the corresponding colors. Statistically non-significant correlations are left blank. The FDR adjusted alpha was α < 0.025 for LS-CTL, α < 0.017 for ACC-CTL, and α < 0.008 for LS-SIS, NAc-CTL, NAc-SIS, and ACC-SIS (Benjamini and Hochberg, 1995).

The data from the home cage observations, body weight measurements, the social approach test, and RT-qPCRs were analyzed using the Grubbs test to detect and remove significant outliers. Home cage observations of behavior (huddling, allo-grooming, social investigation, and aggression) were analyzed with R software (version 4.5.1). Because we were unable to individually identify each animal within a pair during the home cage observations (i.e., pairing periods), the nature of our data was interdependent but the sequence of pairings for each subject was unique. Thus, we analyzed our data using a linear mixed-model (LMM) framework with the packages lme4 (Bates et al., 2015), with fixed effects of Group (CTL vs. SIS) and Pairing Period, and with random effects of subject ID and pair ID. Although the data give the visual impression of pseudoreplication, leveraging an LMM with these random effects included account for this apparent non-independence across the repeated measures, and correctly estimates the variance contributed at each level (see Brown, 2021). Individual analyses were used to compare these behaviors during the Introductory Periods (IP_1–5_), the Familiarity Periods (FP_1–5_), and to compare the change within each pairing period (ΔPP_*n*_ = FP_*n*_ - IP_*n*_). Because subject within the dyads were indistinguishable, subject ID and dyad ID were included as random effects in the LMM for home cage observations. Significant interactions or significant main effects (α = 0.05) were followed by the R package emmeans (Lenth et al., 2022). Body weight, SAT, and RT-qPCR data were tested for normality (Shapiro–Wilk test) and equal variance to compare the CTL and SIS groups by using Prism (version 10.6.1, GraphPad Software, San Diego California United States). When data were found to be normally distributed, an unpaired *t*-test (two-tailed) was performed. If data were not normally distributed, a Mann-Whitney test was performed. Body weight and body weight change were analyzed using an LMM to compare the effects of Group (CTL vs. SIS) and Postnatal Day (PND31 vs. PND41). Behaviors from the social approach test were analyzed to compare the effects of Group (CTL vs. SIS) on social zone duration, latency to approach a stimulus, SI ratio, zone frequency, distance moved, and velocity. The Wilcoxon signed-rank test was used to evaluate SI ratio medians to the hypothetical mean of 1, which is used to define stress susceptible (< 1) and stress resilient (> 1) phenotypes. Figures 3–8 and Supplementary Figures S1, S2 were created using Prism (version 10.6.1, GraphPad Software, San Diego California United States) and all data are presented as the means ± standard error of mean (SEM). We performed Pearson correlations between mRNA gene receptor expression within each group and *p*-values were adjusted for multiple comparisons with False Discovery Rate (FDR) (Benjamini and Hochberg, 1995). The Hmisc and corrplot packages in R were used to visualize the correlograms in Figure 9 and Supplementary Figures S3–S8 (Wei, 2024; Harrell and Dupont, 2025).

## Results

### Home cage observations during the SIS paradigm

We assessed home cage interactions between dyads throughout the SIS paradigm (Figure 1) to determine how huddling, allo-grooming, social investigation, and aggressive behavior would change over time. Home cage observations were recorded during the first 5 min of introducing subjects to one another (Introductory Periods 1–5, IP_1–5_) and during the last 5 min of the 2-day cohabitation (Familiarity Periods 1–5, FP_1–5_), right before each dyad was re-paired (Figure 1). CTL subjects were re-paired with the same litter mate throughout the experimental paradigm. We analyzed IP and FP data separately to determine whether SIS impacted social behavior upon first meetings (IP) or after familiarity had stabilized (FP). We also compared the change in duration for huddling, allo-grooming, social investigation, and aggressive behaviors between the first 5 min of being introduced to a novel conspecific (SIS) or being re-paired with the same conspecific (CTL) and the FP (last 5 min of cohabitating for 2 days with the same conspecific) to determine if the interactions between dyads differed between groups (CTL vs. SIS) and within each pairing period (ΔPP_*n*_ = FP_*n*_ - IP_*n*_).

#### Huddling duration

Huddling duration during the IP significantly differed by Group [SIS v CTL: *F*_(1_, _94)_ = 31.50, *p* = 2.01e-7; Figure 3A], but neither Pairing Period [*F*_(1_, _94)_ = 0.00, *p* = 0.10] nor the interaction between Group and Pairing Period [*F*_(1_, _94)_ = 0.16, *p* = 0.69] were significant. *Post hoc* comparisons showed that the CTL group significantly spent more time huddling than the SIS group during each introductory period (IP_1_: *t*_94_ = 3.50, estimate = 35.0, s.e. = 10.00, *p* = 0.0007; IP_2_: *t*_94_ = 4.73, estimate = 33.4, s.e. = 7.05, *p* < 0.0001; IP_3_: *t*_94_ = 5.62, estimate = 31.7, s.e. = 5.65, *p* < 0.0001; IP_4_: *t*_94_ = 4.40, estimate = 30.1, s.e. = 6.84, *p* < 0.0001; IP_5_: *t*_94_ = 2.94, estimate = 28.5, s.e. = 9.71, *p* = 0.004). Huddling duration during final interactions during the FP did not differ by Group [*F*_(1_, _94)_ = 2.01, *p* = 0.16] or Pairing Period [*F*_(1_, _94)_ = 0.31, *p* = 0.58]. However, we found a significant interaction between Group and Pairing Period [Figure 3B, *F*_(1_, _94)_ = 5.02, *p* = 0.03] for huddling duration. *Post hoc* comparisons showed that the CTL group significantly spent more time huddling than the SIS group during the familiarity periods 4 and 5 (FP_4_: *t*_94_ = 2.45, estimate = 34.94, s.e. = 14.3, *p* = 0.02; FP_5_: *t*_94_ = 2.66, estimate = 53.83, s.e. = 20.3, *p* = 0.009), but not during FP_1–3_ (FP_1_: *t*_94_ = −1.04, estimate = −21.71, s.e. = 20.9, *p* = 0.30; FP_2_: *t*_94_ = −0.19, estimate = −2.83, s.e. = 14.7, *p* = 0.85; FP_3_: *t*_94_ = 1.36, estimate = 16.06, s.e. = 11.8, *p* = 0.18). Lastly, we found that change in huddling duration was not significant for either Group [*F*_(1_, _94)_ = 1.56, *p* = 0.21] or Pairing Period [*F*_(1_, _94)_ = 0.30, *p* = 0.58]. However, the Group by Pairing Period interaction was significant [*F*_(1_, _94)_ = 5.68, *p* = 0.02; Figure 3C]. Overall, subjects in both groups tended to spend more time huddling with their conspecific toward the end of the pairing period (once they became familiar), producing positive mean ΔPP values. Our *post hoc* comparisons showed that the SIS group increased huddling durations more than the CTL group during the first pairing period (*t*_94_ = −2.66, estimate = −56.7, s.e. = 21.3, *p* = 0.009) and second pairing period (*t*_94_ = −2.41, estimate = −36.2, s.e. = 15.0, *p* = 0.018). Huddling duration did not differ between SIS and CTL for pairing periods 3–5 (ΔPP_3_: *t*_94_ = −-1.30, estimate = −15.7, s.e. = 12.0, *p* = 0.20; ΔPP_4_: *t*_94_ = 0.33, estimate = 4.80, s.e. = 14.6, *p* = 0.74; ΔPP_5_: *t*_94_ = 1.22, estimate = 25.3, s.e. = 20.7, *p* = 0.22).

Taken together, these data indicate that SIS animals engaged in less huddling overall, particularly during the introductory periods, which is to be expected for animals that are less familiar with each other. Notably, the difference in huddling between SIS and CTL males at the end of a pairing period (when familiarity should have been established) was only different in the fourth and final paring periods, suggesting that SIS males demonstrate less forms of physical attachment only after several bouts of instability. Similarly, the change in huddling within a pairing period, after 2 days of co-habituation with a new conspecific, was greatest in SIS males but only within the first two pairing periods, consistent with the idea that repeated rounds of social instability eliminated this increase huddling over a pairing period.

#### Allo-grooming duration

Like huddling duration, allo-grooming during the IP significantly differed by Group [*F*_(1_, _94)_ = 7.59, *p* = 0.007; Supplementary Figure S1A], but neither Pairing Period [*F*_(1_, _94)_ = 2.54, *p* = 0.11] nor the interaction between Group and Pairing Period [*F*_(1_, _94)_ = 2.61, *p* = 0.11] were significantly different. *Post hoc* comparisons showed that the CTL group significantly spent more time allo-grooming than the SIS group during introductory periods 1 and 2 (IP_1_: *t*_94_ = 2.91, estimate = 9.61, s.e. = 3.30, *p* = 0.004; IP_2_: *t*_94_ = 3.21, estimate = 7.46, s.e. = 2.32, *p* = 0.002). The SIS groups significantly spent more time allo-grooming than the CTL group during the Introductory Period 3 (IP_3_: *t*_94_ = 2.85, estimate = 5.31, s.e. = 1.86, *p* = 0.005). The CTL and SIS groups did not differ in time spent allo-grooming during introductory periods 4 and 5 (IP_4_: *t*_94_ = 1.40, estimate = 3.16, s.e. = 2.26, *p* = 0.17; IP_5_: *t*_94_ = 0.31, estimate = 1.00, s.e. = 3.20, *p* = 0.75). Allo-grooming duration during the Familiarity Period did not differ by Group [Supplementary Figure S1B; *F*_(1_, _94)_ = 1.09, *p* = 0.30], Pairing Periods [*F*_(1_, _94)_ = 0.31, *p* = 0.58], or an interaction between Group and Pairing Periods [*F*_(1_, _94)_ = 0.23, *p* = 0.63]. Lastly, the duration of allo-grooming did not change over each pairing period resulting in values close to zero for both groups across all pairing periods (Supplementary Figure S1C). Our analysis demonstrated that change in allo-grooming duration did not significantly differ by Group [*F*_(1_, _94)_ = 0.31, *p* = 0.58], Pairing Period [*F*_(1_, _94)_ = 1.62, *p* = 0.21] nor the Group × Pairing Period interaction [*F*_(1_, _94)_ = 1.50, *p* = 0.22]. Altogether, these data indicate that the SIS group engaged in less allo-grooming only during the first two Introductory Periods. Only 1 SIS pair and no CTL animals exhibited allo-grooming during IP_3_. The difference in allo-grooming between SIS and CTL males during IP_4_, IP_5_, and all Familiarity Periods did not differ, suggesting that SIS males demonstrate less forms of affiliation only after initial exposure to social instability.

#### Social investigation

Social investigation during initial interactions (Introductory Period) significantly differed by Group [*F*_(1_, _94)_ = 114.30, *p* < 2.00e-16; Figure 4A], whereas neither Pairing Period [*F*_(1_, _94)_ = 0.07, *p* = 0.79] nor the interaction between Group and Pairing Period [*F*_(1_, _94)_ = 3.15, *p* = 0.08] were significant. *Post hoc* comparisons showed that the SIS group significantly spent more time socially investigating their conspecifics than the CTL group during the initial interactions 1–5 (IP_1_: *t*_94_ = −4.57, estimate = −45.2, s.e. = 9.90, *p* < 0.0001; IP_2_: *t*_94_ = −7.50, estimate = −52.3, s.e. = 6.97, *p* = ; IP_3_: *t*_94_ = −10.62, estimate = −59.4, s.e. = 5.59, *p* < 0.0001; IP_4_: *t*_94_ = −9.81, estimate = −66.5, s.e. = 6.77, *p* < 0.0001; IP_5_: *t*_94_ = −7.652, estimate = −73.5, s.e. = 9.61, *p* < 0.0001). Social investigation during final interactions (Familiarity Period) also significantly differed by Group [Figure 4B, *F*_(1_, _94)_ = 56.28, *p* = 3.49e-11] but not Pairing Period [*F*_(1_, _94)_ = 3.22, *p* = 0.08], and the interaction between Group and Pairing Period [*F*_(1_, _94)_ = 2.67, *p* = 0.11] was not significant. *Post hoc* comparisons showed that the SIS group significantly spent more time socially investigating their conspecifics than the CTL group during the familiarity periods 1–5 (FP_1_: *t*_94_ = −2.91, estimate = −5.57, s.e. = 1.91, *p* = 0.004; FP_2_: *t*_94_ = −5.07, estimate = −6.83, s.e. = 1.35, *p* < 0.0001; FP_3_: *t*_94_ = −7.50, estimate = −8.09, s.e. = 1.08, *p* < 0.0001; FP_4_: *t*_94_ = −7.16, estimate = −9.35, s.e. = 1.31, *p* < 0.0001; FP_5_: *t*_94_ = −5.72, estimate = −10.61, s.e. = 1.85, *p* < 0.0001). Finally, social investigation generally decreased over each pairing period resulting in negative values for both groups across all pairing periods. Our analysis demonstrated that change in social investigation duration significantly differed by Group [*F*_(1_, _94)_ = 86.91, *p* = 5.07e-15; Figure 4C], but neither the Pairing Period [*F*_(1_, _94)_ = 0.38, *p* = 0.54] nor the Group × Pairing Period interaction [*F*_(1_, _94)_ = 2.16, *p* = 0.14] were significantly different. *Post hoc* comparisons showed that the SIS group significantly reduced social investigation more (i.e., had a greater negative change in social investigation duration) than the CTL group for each pairing period (ΔPP_1_: *t*_94_ = 4.04, estimate = 39.6, s.e. = 9.81, *p* = 0.0001; ΔPP_2_: *t*_94_ = 6.57, estimate = 45.4, s.e. = 6.91, *p* < 0.0001; ΔPP_3_: *t*_94_ = 9.25, estimate = 51.3, s.e. = 5.54, *p* < 0.0001; ΔPP_4_: *t*_94_ = 8.51, estimate = 57.1, s.e. = 6.71, *p* < 0.0001; ΔPP_5_: *t*_94_ = 6.60, estimate = 62.9, s.e. = 9.53, *p* < 0.0001).

Collectively, these data indicate that SIS showed high levels of initial social investigation with a novel conspecific upon the introduction to a new conspecific and at the conclusion of the pairing period, whereas CTL animals consistently investigated their litter mates less, presumably reflecting differences in familiarity within and between pairing periods. Also supporting this interpretation, SIS decreased investigation relatively more than CTL males within pairing periods, potentially because CTL males simply engaged in less social investigation overall.

#### Aggression duration

Consistent with other studies, our non-bonded males did not demonstrate high levels of aggressive behavior regardless of social stability or instability. The data set for the duration of aggression exhibited a high proportion of zero values, which violated the assumption of normality required for a linear mixed model. Consequently, a quantitative analysis using traditional methods was not appropriate, and the findings are presented through a detailed qualitative interpretation. For descriptive purposes, we present the duration of aggression (Figure 5) like the figures for the duration of huddling and social investigation. Notably, only SIS males engaged in aggression, and this only happened in the introductory periods of 1, 4, and 5 (Figure 5A). Only one dyad engaged in aggression during IP_1_ (composed of subjects SIS-4 + SIS-5). Two dyads engaged in aggression during IP_4_ (SIS-2 + SIS-5 and SIS-4 + SIS-8). Finally, one dyad engaged in aggression during IP_5_ (SIS-7 + SIS-10). No CTL or SIS subjects displayed aggression during the familiarity period (Figure 5B) and therefore the change in pairing period (Figure 5C) mirrored the introductory period. It is notable that the same animals that were paired together in pairing period 1, also demonstrated aggression with other conspecifics in pairing period 4, but an entirely new dyad of animals demonstrated the aggression in the 5*^th^* pairing period. Therefore, the extent to which aggression can be directly attributed to one or two specific animals is unclear. We also note that although aggression was low overall, it did appear to marginally increase in later pairing periods (see marginal means presented in Figure 5), possibly indicating that aggression becomes more likely following multiple rounds of social instability. However, even though this is consistent with the data presented above on huddling, this conclusion should be taken with caution considering the qualitative and limited nature of the aggression data.

### Body weight and body weight gain

Subjects were weighed right before the Introductory Period (IP) of each pairing period (PND31, 33, 35, 37, 39, and 41) to approximate body size changes between groups and across the experiment. Not surprisingly, we found that body weight significantly increased over time [Postnatal Day; *F*_(5_, _108)_ = 5.440, *p* = 1.673e-4; Figure 6A]. However, body weight did not differ by Group [*F*_(1_, _108)_ = 0.967, *p* = 0.328] and the interaction between Group × Postnatal Day [*F*_(5_, _108)_ = 0.056, *p* = 0.998] was not significant. Overall body weight gain (PND41 body weight −- PND31 body weight) also did not differ between groups (unpaired *t-*test: *p* = 0.871, t_18_ = 0.165; Figure 6B). Thus, despite some behavioral differences noted above, social instability stress did not appear to impact our proxy for physical development in male prairie voles.

### Social approach behaviors and stress phenotypes

One animal tested during the SAT never entered the social zone during the habituation phase, and therefore we were unable to calculate an SI ratio for this subject. Furthermore, the Grubbs test indicated that one animal from the SIS group was a significant outlier and should be removed from the social latency comparison, and one animal from each group was a significant outlier and should be removed from the SI ratio comparison. Thus, our sample sizes ranged from *N* = 8 to 10 (see Figure 7). Subjects spent similar amounts of time in the social zone during the SAT regardless of group (Mann Whitney test: *p* = 0.352; Figure 7A). Subjects also did not differ in the latency to approach a stimulus in the SAT (Mann Whitney test: *p* = 0.063; Figure 7B), although there was a non-significant trend for CTL animals to take longer to approach the stimulus animal. The frequency of entries into the social zone was also not significantly different between groups (unpaired *t-*test: t_18_ = 0.762, *p* = 0.456; Supplementary Figure S2A). Similarly, the distance moved (t_18_ = 0.346, *p* = 0.733; Supplementary Figure S2B) and velocity of movement (t_18_ = 0.346, *p* = 0.733; Supplementary Figure S2C) were not significantly different between groups. These results indicate that the SIS subjects were just as likely as CTL subjects to approach and spend time near the vicinity of a novel conspecific.

Notably, the duration of time that CTL subjects spent in the social zone significantly (negatively) correlated with the latency to approach a stimulus (*r* = −0.78, *p* = 7.41e-3; Supplementary Table 2) whereas SIS subjects did not show this relationship (*r* = −0.02, *p* = 0.97). Furthermore, the SI ratios (a metric of stress resiliency or susceptibility) between groups significantly differed (Mann Whitney test: *p* = 0.045; Figure 7C) with SIS males demonstrating greater SI ratios than CTL males. Comparing the SI ratio to 1 provides a threshold for defining stress phenotypes (<1 = stress susceptible; greater than 1 = stress resilient). Males in the SIS group showed a median SI ratio that was significantly greater than 1 (Wilcoxon signed-rank test: *p* = 0.008; Figure 7C), indicating that SIS males were categorically “stress resilient.” In contrast, males assigned to the CTL group showed a range of variation in their SI ratios (both above and below 1) such that the median SI ratio for the CTL group did not differ from 1 (*p* = 0.340). Such a range in natural variation of the SI ratio is expected in non-manipulated animals.

### Gene receptor expression and coordinated expression

We assessed the impact of social instability stress on *Oxtr*, *Avpr1a*, *Drd1*, and *Drd2* gene expression in the lateral septum, nucleus accumbens, and anterior cingulate cortex in tissue collected immediately after the SAT. The Grubbs test indicated that one subject’s measurements within the SIS or CTL group was a significant outlier and should be removed for some of the gene comparisons within the LS, NAc, and/or ACC (see Supplementary Table 3 for final sample sizes). Although observing that CTL and SIS groups differed behaviorally, we found no significant differences in receptor gene expression in the LS, NAc, and ACC (Figure 8 and Supplementary Table 3).

Correlations between gene expression and behavior (both within and between groups) have the potential to reveal patterns of gene-behavior correspondences that might reflect functional differences in brain processing that result from different experiences and shape individual differences in behavior. Therefore, we next correlated gene receptor expression of each group with SAT social zone duration, latency to approach a stimulus, and SI ratio. Although *Avpr1a* expression did not differ between groups (see Figure 8B), *Avpr1a* expression in the LS of CTL subjects, but not SIS subjects, negatively correlated with the latency to approach a stimulus during the SAT (CTL: *r* = 0.70, *p* = 2.44e-2; SIS: *r* = 0.02, *p* = 0.96; Supplementary Table 4). Conversely, LS-*Drd1* and LS-*Drd2* expression negatively correlated with the SI ratios of SIS subjects (SI ratio vs. LS-*Drd1*: *r* = −0.81, *p* = 0.03; SI ratio vs. LS- *drd2*: *r* = −0.97, *p* = 9.00e-5) but not CTL subjects (SI ratio vs. LS-*Drd1*: *r* = 0.17, *p* = 0.67; SI ratio vs. LS-*Drd2*: *r* = 0.26, *p* = 0.50). In the NAc, the social zone duration of CTL subjects negatively correlated with *Drd2* expression (CTL: *r* = −0.67, *p* = 4.96e-2; SIS: *r* = −0.52, *p* = 0.12; Supplementary Table 4). Lastly, the correlation between ACC- *Drd2* expression and the latency to approach a stimulus were in opposing directions for CTL (*r* = −0.67, *p* = 4.79e-2) and SIS (*r* = 0.79, *p* = 0.02) subjects. All other correlations between gene receptor expression and SAT behavior were not significant.

Particular life experiences have been known to induce a concordance of gene expression among the oxytocin, vasopressin, and dopamine signaling systems, such that animals exposed to one form of experience show coordinated gene expression patterns whereas another experience might lead to non-coordinated gene expression (Forero et al., 2024; Sailer et al., 2022b). We therefore assessed correlations between gene receptor expression within SIS and CTL males to determine if there was a pattern of coordinated gene expression among *Oxtr*, *Avpr1a*, *Drd1*, and *Drd2* within the LS, NAc, and ACC for either group. Interestingly, several discrete positive correlations between mRNA expression were observed following an FDR adjustment for multiple comparisons (Benjamini and Hochberg, 1995; Figure 9). CTL and SIS subjects demonstrated positive correlations between *Drd1* and *Drd2* within the LS (CTL: *r* = 0.95, *p* = 2.67e-5, FDR α < 0.025, Figure 9A and Supplementary Figure S3; SIS: *r* = 0.85, *p* = 0.003, FDR α < 0.008, Figure 9B and Supplementary Figure S4) and the NAc (CTL: *r* = 0.83, *p* = 0.006, FDR α < 0.008, Figure 9C and Supplementary Figure S5; SIS: *r* = 0.93, *p* = 0.0001, FDR α < 0.008, Figure 9D and Supplementary Figure S6) but not in the ACC (CTL: *r* = 0.67, *p* = 0.05, FDR α < 0.017, Figure 9E and Supplementary Figure S7; SIS: *r* = 0.31, *p* = 0.41, FDR α < 0.008, Figure 9F and Supplementary Figure S8). Notably, we highlight the positive correlations between LS gene expression of *Oxtr* and *Drd1* (*r* = 0.81, *p* = 4.65e-3, FDR α < 0.025) and between *Oxtr* and *Drd2* (*r* = 0.93, *p* = 1.02e-4, FDR α < 0.025) of CTL subjects, which were not found in the LS of SIS subjects. Similarly, these relationships were observed in the ACC of CTL subjects (*Oxtr* and *Drd1*: *r* = 0.85, *p* = 0.003, FDR α < 0.017; *Oxtr* and *Drd2*: *r* = 0.79, *p* = 0.01, FDR α < 0. 017), but not in the ACC of SIS subjects. Taken together, these data indicate that social experience, regardless of familiarity with a social partner, positively coordinates LS and NAc co-expression of *Drd1* and *Drd2*. Moreover, and perhaps more interestingly, stable and long-term interactions with a familiar conspecific (e.g., a sibling) uniquely coordinates co-expression of *Oxtr* and *Drd1* and *Oxtr* and *Drd2* in the LS and ACC.

## Discussion

Adolescence represents a critical window during which genes and neural circuits that mediate social motivation and stress responsivity undergo substantial maturation (Andersen and Teicher, 2008; Tottenham and Galvan, 2016). The present study examined how adolescent social instability stress, induced under an ecologically relevant testing paradigm, affects social behaviors with novel and familiar conspecifics and patterns of gene expression for receptors that bind signaling molecules central to modulation of social behavior (oxytocin, vasopressin and dopamine) in male prairie voles. Consistent with prior research, social instability stress disrupted affiliation and may have facilitated aggression under typical housing conditions, potentially reflecting altered oxytocin, arginine vasopressin, and dopamine signaling within limbic structures that regulate social attachment (Lim and Young, 2006; Lieberwirth and Wang, 2014). However, in contrast to stress paradigms that produce generalized social withdrawal (Golden et al., 2011; Kelly et al., 2020; Sailer et al., 2022b), SIS subjects displayed sustained social approach and investigative behaviors, suggesting an adjustment of social reward and stress-coping systems rather than global social suppression. This pattern parallels reports that moderate chronic social stress can facilitate behavioral flexibility and resilience through alterations in dopaminergic signaling within the nucleus accumbens and prefrontal cortex (Russo et al., 2012; Walker et al., 2017; Diaz and Lin, 2020). Our results go beyond simple group differences in gene expression and highlight the importance of considering the coordinated expression of a suite of genes, and how this might relate to patterns of behavior within and between groups of animals.

### Disruption of affiliation

Our findings reveal that repeated episodes of social instability disrupted affiliative behavior (i.e., huddled less) during home cage observations after subjects had the opportunity to become familiar with a novel conspecific. Interestingly huddling did not differ for the first few pairing periods after familiarity was established. However, as the impacts of social instability took hold, it appeared that huddling became comparatively less common among SIS males. These data suggest that repeated exposure to social instability reduced the probability that males would engage in prosocial huddling behaviors, potentially reflecting a “dampening” of their willingness to engage in affiliative behaviors. Male mice are known to engage in social withdrawal after SIS exposure (Yohn et al., 2019). Although social huddling might be a less ecologically relevant behavior in mice than it is in prairie voles, the reduction in huddling behavior is certainly consistent with the idea that male prairie voles became socially withdrawn over time with SIS exposure. Indeed, social instability in childhood has frequently been linked with social withdrawal in humans (Oh et al., 2008), suggesting a broad causal link between social instability and affiliation.

Moreover, anecdotal evidence suggested that social instability might have also facilitated aggressive behavior during first encounters with a new conspecific. Aggression was very rare and only exhibited by SIS subjects. Because the incidence of aggression was so uncommon, it was not possible to statistically assess if duration of aggression differed between SIS and CTL subjects. Of the few instances of aggression, we only observed aggression during the introductory period (and not during familiarity periods), and it was primarily observed after animals had experienced several rounds of social instability. These qualitative results are consistent with other work that has indicated that social instability often leads to increased observations of aggression (Willcox et al., 2025). Across taxa, social instability has been tied to increased stress responsivity, and glucocorticoid release more specifically (Maldonado-Chaparro et al., 2025), where higher levels of glucocorticoids reflect responses to unstable environments. Relatively acute exposure to endogenous glucocorticoid release, like that which is expected to occur during the first few moments of a social interaction with an unfamiliar conspecific, have been linked to increases in aggressive responses (Haller, 2022). However, these observations do not necessarily reflect generalized stress priming or diminished stress resilience. Instead, they may indicate context-dependent social reactivity that is specific to initial encounters with unfamiliar conspecifics. Such reactivity may occur alongside behavioral patterns consistent with stress resilience, including enhanced social approach behaviors and recalibration of physical social behaviors the support continued social engagement in SIS males. Nevertheless, the relative infrequent incidences of aggression that we observed merits caution when considering this interpretation.

### Social investigation vs. social approach

All subjects were more investigative of their paired conspecifics in the early stages of each pairing period. After a 2-day cohabitation period, near the end of each pairing period, social investigation continued to be stable, however—not surprisingly—subjects were more investigative at the beginning than the end of each pairing period. Notably, SIS males experiencing social instability consistently exhibited higher levels of social investigation compared to CTL subjects both within and between pairing periods. We interpret this difference to indicate a typical response to conspecific novelty (SIS) or familiarity (CTL). The consistent levels of investigation of SIS males contrasted with the reduction of affiliation and anecdotal increase in aggression over the course of repeated social re-pairings. This indicates some degree of domain specificity for how social instability impacts information gathering (or perhaps social curiosity) compared to other kinds of social behaviors (like attraction and aversion) that are more sensitive to social instability.

Although we found that SIS subjects spent more time investigating their cage-mates than CTL subjects during the re-pairing regimen in the home cage, SIS and CTL subjects exhibited similar patterns of social approach toward novel conspecifics (latency and frequency to enter, and total time spent in the social zone) during the social approach test, potentially reflecting nuanced differences between the contextual function of social investigation and social approach. This apparent discrepancy between observations of social investigation in the home cage and social approach during the SAT can be explained by the distinction between three related but separable components of rodent social behavior: approach, investigation, and affiliation. Social approach reflects the initial motivational process of engaging with a conspecific, a behavior often driven by curiosity, vigilance, or general sociability (Sailer et al., 2022a). Once proximity is achieved, rodents engage in social investigation, such as sniffing the facial and anogenital regions. This component of social behavior serves to gather information about sex, reproductive status, and familiarity, and is necessary when rodents encounter unfamiliar conspecifics (Ophir et al., 2009). Social affiliation encompasses the maintenance of close, reciprocal contact, and is a hallmark of stable social bonding in prairie voles. Social affiliation is strongly modulated by familiarity, as rodents are more likely to maintain affiliative contact with familiar conspecifics than with strangers (Kelly et al., 2018). Accordingly, SIS voles may retain normal motivation to approach and investigate both familiar and unfamiliar conspecifics, consistent with curiosity, vigilance, or general sociability, yet show reduced motivation to transition these exploratory interactions into the sustained physical contact characteristic of social affiliation when compared to CTL subjects. Viewed through this framework, adolescent SIS may enhance investigative vigilance at the expense of extended affiliative contact with familiar conspecifics and creates a context-specific pattern that aligns logically once the distinct components of social behavior are considered.

### Does social disruption facilitate stress resilience?

Despite the apparent lack of behavioral differences between SIS and CTL males in the SAT, SIS voles exhibited SI ratios > 1.0, reflecting a characteristically stress-resilient phenotype. This result adds support to the hypothesis that adolescent social instability stress may shift, rather than suppress, social motivation in male prairie voles. Interestingly, the impact of SIS on emotional behavior in other rodents is not uniform (Mccormick and Green, 2013; Mccormick et al., 2015; Koert et al., 2021). Many studies report that SIS causes anxiety-like behaviors (Mccormick and Green, 2013). However, some evidence shows the opposite; for example, adolescent SIS has been reported to have no effects on male rat anxiety-like behavior (Mccormick et al., 2008). Such findings indicate that social instability stress can influence emotional and motivational systems in multiple directions, depending on developmental timing and context. The stress resilience phenotype observed in SIS subjects may reflect a shift in how social cues are processed after repeated social instability, resulting in preserved approach and investigation with unfamiliar conspecifics. Indeed, the compression of social resilience among SIS subjects lends credence to the adage: “*What doesn’t kill us makes us stronger.*” Alternatively, considering that our measure of social resiliency is based on time near vs. time far from an unfamiliar conspecific, this compression of social resiliency among SIS males could simply reflect the facilitation of a form of “social curiosity” of strangers.

### Patterns of gene expression reveal deeper insight into behavioral output

Finally, our results failed to show a direct relationship between SIS and gene expression of oxytocin receptor, arginine vasopressin receptor 1a, or D1-like and D2-like dopamine receptors. Nevertheless, our results did reveal some notable patterns of gene expression coordination among control animals, and comparatively less gene expression coordination among SIS males within the LS and ACC. These results could indicate that within group variation in the latency to social approach and the time spent investigating a novel conspecific might relate to the degree of gene expression coordination between D1R, D2R and OTR in the LS and OTR with D1R and D2R in the ACC.

Even with clear behavioral differences between groups, we did not observe significant group-level differences in *Oxtr*, *Avpr1a*, *Drd1*, or *Drd2* mRNA expression within the LS, NAc, or ACC. This lack of mean differences suggests that adolescent SIS may not produce widespread transcriptional changes, but instead marginally alter the functional associations and coordinated activity among the oxytocin, vasopressin, and dopamine systems. Supporting this interpretation, several region-specific and receptor-specific correlations emerged between behavioral responses and gene expression. Expression of *Avpr1a* in the LS, a hub for integrating social salience and emotional state (Sheehan et al., 2004) negatively correlated with the latency of CTL subjects to approach a novel conspecific during the SAT. This finding is consistent with the established role of vasopressin signaling in promoting social investigation with novel conspecifics (Rigney et al., 2024; Lukas et al., 2011) and reducing social investigation with familiar conspecifics (Lukas et al., 2011). Interestingly, this relationship was absent in SIS subjects, suggesting that social instability stress disrupted the functional link between septal vasopressin signaling and social approach behavior. Conversely, LS *Drd1* and *Drd2* expression in SIS subjects negatively correlated with SI ratios, a pattern not seen in controls. These findings imply that elevated dopamine receptor expression in the LS of SIS subjects may relate to a context-dependent social strategy, consistent with the notion that enhanced dopaminergic signaling can facilitate social selectivity and stress-coping following a social challenge (Aragona et al., 2006; Campi et al., 2014). The NAc dopamine system may contribute to species-specific social organization in prairie voles, as D1R (encoded by *Drd1*) and D2R (encoded by *Drd2*) activation can differentially enable the formation and maintenance of pair bonds (Aragona et al., 2006). Social defeat stress caused a greater social withdrawal that was linked to increased NAc-D1R activation and this pattern was reversed by D1R antagonism (Campi et al., 2014). In the NAc and ACC of CTL subjects, SAT social zone duration negatively correlated with *Drd2* expression, suggesting that social instability stress may reconfigure prefrontal and limbic brain nodes that modulate reward-stress integration to enable social approach in unfamiliar contexts.

Analysis of gene receptor expression correlations revealed that SIS in male prairie voles altered the coordinated expression patterns of the oxytocin, vasopressin, and dopamine systems across regions. Both groups showed positive correlations between *Drd1* and *Drd2* expression within the LS and NAc, suggesting preserved co-regulation of dopaminergic signaling under stress and control conditions. However, CTL subjects exhibited strong positive associations between *Oxtr* and dopamine receptor expression (*Oxtr-Drd1* and *Oxtr-Drd2*) in the LS and ACC, which are absent in SIS subjects. This may suggest that adolescent social instability stress can disrupt the functional integration between oxytocin and dopamine signaling pathways that normally facilitate social reward and attachment. In prairie voles, oxytocin and dopamine interactions within the NAc and prefrontal regions are critical for the formation and maintenance of selective social bonds (Liu and Wang, 2003). The absence of such coordinated expression in SIS voles may bring about their reduced affiliative behavior, despite continued social investigation and approach behaviors, suggesting that social instability stress could weaken the neurochemical coupling that reinforces the rewarding aspects of social contact.

Together, these findings indicate that adolescent male SIS does not produce global suppression of social behavior but rather contributes to a context-dependent modulation of social behavior and the underlying functional architecture of the nonapeptide and dopaminergic systems. The dissociation between sustained social approach and social investigation coupled with disrupted social affiliation points to a context-dependent reorganization of social motivation. Rather than reflecting a deficit, this pattern may reflect a compensatory shift in social motivation: a shift in how social cues are processed and rewarded to optimize behavior in environments characterized by social instability. Such recalibration may involve modulation of oxytocin–dopamine interactions within the LS and ACC, brain regions critical for integrating social reward and stress reactivity. This interpretation aligns with the stress inoculation framework, which posits that mild or predictable stressors during development can promote resilience by fine-tuning neurocircuitry involved in emotional regulation and social engagement across different species (Lyons and Parker, 2007; Ayash et al., 2020; Edge et al., 2009). Accordingly, the behavioral resilience observed in SIS male subjects may emerge from plasticity of the social reward circuit, which might support social engagement but not social affiliation.

Although mRNA expression provides valuable insights into potential receptor regulation, future studies should measure receptor protein density or use pharmacology to co-block receptors to confirm whether transcriptional coordination corresponds to functional interactions and behavioral responses. Additionally, our study focused on male responses to SIS, leaving the question of how females might react unanswered. Therefore, investigating sex differences and extending behavioral assessment into adulthood will clarify whether these behavioral molecular alterations persist across development. Finally, linking receptor expression patterns to markers of recent neural activation (e.g., via immediate-early gene expression) during social behavioral interactions could elucidate how SIS modifies real-time circuit engagement during familiar and unfamiliar social contexts.

In summary, the present study demonstrates that adolescent SIS alters social behavior and disrupts the coordination of the nonapeptide and dopaminergic signaling systems in male prairie voles. Although subjects that experienced SIS exhibited reduced affiliative behaviors in familiar contexts, they maintained high degrees of social approach and exhibited behavioral markers of resilience, suggesting SIS induced a social domain recalibration rather than global social impairment. The absence of significant mRNA expression of *Oxtr*, *Avpr1a*, *Drd1*, and *Drd2* between groups, combined with altered patterns of receptor-behavior and receptor co-expression correlations, indicates that SIS influences the integration and functional balance of oxytocin, vasopressin, and dopamine pathways within key limbic regions of the brain. These findings underscore the importance of considering network-level plasticity, rather than isolated molecular changes, in understanding how early social adversity shapes social motivation and stress coping. By extending the SIS paradigm to a socially complex species, this work provides a framework for exploring how developmental social environments sculpt the neurobiology of attachment and resilience across species, including humans.

## Data Availability

The raw data supporting the conclusions of this article will be made available by the authors, without undue reservation.
