# Supplementary material for: Adolescent social instability stress alters social processes in male prairie voles
Source: Front Behav Neurosci. 2026 Feb 24;20:1761549. doi: 10.3389/fnbeh.2026.1761549 (PMC12971939; doi:10.3389/fnbeh.2026.1761549)
Supplement: Supplementary file 1 [file Data_Sheet_1.docx]

**Supplemental Table 1 | Primer sequences used for qPCR.**

| **Gene target** | **Sequence (5’🡪3’)** |
| --- | --- |
| ***Oxtr*** | **For:** TCCAAGGCCAAAATCCGCACGG |
|  | **Rev:** GGCAGAAGCTTCCTTGGGCGC |
| ***Avpr1a*** | **For:** GAGGTGAACAATGGCACTAAAACC |
|  | **Rev:** CCAGATGTGGTAGCAGATGAAGC |
| ***Drd1*** | **For:** TTAACAACAATGGGGCTGTG |
|  | **Rev:** GGCATGAGGGATCAGGTAAA |
| ***Drd2*** | **For:** GTGAAGGCGCTGTAGAGGAC |
|  | **Rev:** CGGTGTGTTCATCATCTGCT |
| ***Nadh*** | **For:** CTATTAATCCCCGCCTGACC |
|  | **Rev:** GGAGCTCGATTTGTTTCTGC |

**Supplemental Table 2 | Correlations between SAT behavior data**

| **Group** | **Behavior** | **r** | ***p*** |
| --- | --- | --- | --- |
| **CTL** | **Social Zone vs Latency** | **-0.78** | ****0.01** |
|  | **Social Zone vs SI Ratio** | -0.25 | 0.52 |
|  | **Latency vs SI Ratio** | 0.11 | 0.79 |
| **SIS** | **Social Zone vs Latency** | -0.02 | 0.97 |
|  | **Social Zone vs SI Ratio** | -0.22 | 0.61 |
|  | **Latency vs SI Ratio** | -0.63 | 0.13 |

**Supplemental Table 3 | Gene receptor expression compared between CTL and SIS groups (unpaired t-tests statistics)**

| **Brain region** | **Gene** | **n/group** | | **t** | **df** | ***p*** |
| --- | --- | --- | --- | --- | --- | --- |
|  |  | **CTL** | **SIS** |  |  |  |
| **LS** | ***Oxtr*** | 10 | 9 | 0.01 | 17 | 0.99 |
|  | ***Avpr1a*** | 10 | 10 | 0.80 | 18 | 0.44 |
|  | ***Drd1*** | 10 | 9 | 0.96 | 17 | 0.35 |
|  | ***Drd2*** | 10 | 10 | 0.93 | 18 | 0.37 |
| **NAc** | ***Oxtr*** | 9 | 10 | 1.46 | 17 | 0.16 |
|  | ***Avpr1a*** | 9 | 10 | 1.11 | 17 | 0.28 |
|  | ***Drd1*** | 9 | 10 | 0.70 | 17 | 0.49 |
|  | ***Drd2*** | 9 | 10 | 0.93 | 17 | 0.37 |
| **ACC** | ***Oxtr*** | 9 | 10 | *1.98* | *17* | *0.06* |
|  | ***Avpr1a*** | 9 | 10 | 0.03 | 17 | 0.97 |
|  | ***Drd1*** | 10 | 10 | 0.19 | 18 | 0.85 |
|  | ***Drd2*** | 9 | 9 | 0.71 | 16 | 0.49 |

**Supplemental Table 4 | Gene receptor expression correlations with SAT behavior data**

| **Brain region** | **Group** | **Gene** | **Social Zone** | | **Latency** | | **SI Ratio** | |
| --- | --- | --- | --- | --- | --- | --- | --- | --- |
|  |  |  | **r** | ***p*** | **r** | ***p*** | **r** | ***p*** |
| **LS** | **CTL** | ***Oxtr*** | 0.23 | 0.53 | 0.03 | 0.94 | 0.40 | 0.29 |
|  |  | ***Avpr1a*** | -0.38 | 0.28 | **0.70** | **0.02** | -0.20 | 0.61 |
|  |  | ***Drd1*** | 0.16 | 0.66 | 0.03 | 0.93 | 0.16 | 0.67 |
|  |  | ***Drd2*** | 0.16 | 0.65 | 0.08 | 0.83 | 0.26 | 0.50 |
|  | **SIS** | ***Oxtr*** | 0.05 | 0.89 | -0.02 | 0.96 | -0.63 | 0.13 |
|  |  | ***Avpr1a*** | 0.49 | 0.15 | 0.02 | 0.96 | -0.40 | 0.32 |
|  |  | ***Drd1*** | 0.33 | 0.38 | 0.40 | 0.32 | **-0.81** | ***0.03** |
|  |  | ***Drd2*** | 0.13 | 0.73 | 0.50 | 0.17 | **-0.97** | ***9.00E-05** |
| **NAc** | **CTL** | ***Oxtr*** | -0.23 | 0.55 | 0.23 | 0.55 | 0.06 | 0.89 |
|  |  | ***Avpr1a*** | 0.07 | 0.85 | -0.01 | 0.97 | 0.30 | 0.46 |
|  |  | ***Drd1*** | *-0.60* | *0.09* | 0.37 | 0.32 | -0.19 | 0.65 |
|  |  | ***Drd2*** | **-0.67** | ***0.0496** | 0.48 | 0.20 | -0.53 | 0.17 |
|  | **SIS** | ***Oxtr*** | -0.31 | 0.38 | 0.18 | 0.65 | 0.13 | 0.76 |
|  |  | ***Avpr1a*** | 0.28 | 0.44 | 0.56 | 0.12 | -0.05 | 0.91 |
|  |  | ***Drd1*** | -0.63 | 5.29E-02 | -0.20 | 0.61 | 0.06 | 0.89 |
|  |  | ***Drd2*** | -0.52 | 0.12 | 2.72E-03 | 0.99 | -0.10 | 0.82 |
| **ACC** | **CTL** | ***Oxtr*** | 0.47 | 0.20 | -0.45 | 0.23 | 0.25 | 0.56 |
|  |  | ***Avpr1a*** | -0.37 | 0.33 | 0.14 | 0.73 | -0.27 | 0.52 |
|  |  | ***Drd1*** | 0.19 | 0.60 | -0.21 | 0.55 | 0.21 | 0.58 |
|  |  | ***Drd2*** | 0.40 | 0.29 | **-0.67** | ***0.048** | 0.05 | 0.90 |
|  | **SIS** | ***Oxtr*** | 0.48 | 0.16 | 0.12 | 0.75 | 0.31 | 0.46 |
|  |  | ***Avpr1a*** | -0.03 | 0.94 | 0.24 | 0.53 | 0.47 | 0.24 |
|  |  | ***Drd1*** | *0.58* | *0.08* | 0.20 | 0.61 | -0.20 | 0.64 |
|  |  | ***Drd2*** | 0.07 | 0.86 | **0.79** | ***0.02** | -0.57 | 0.18 |


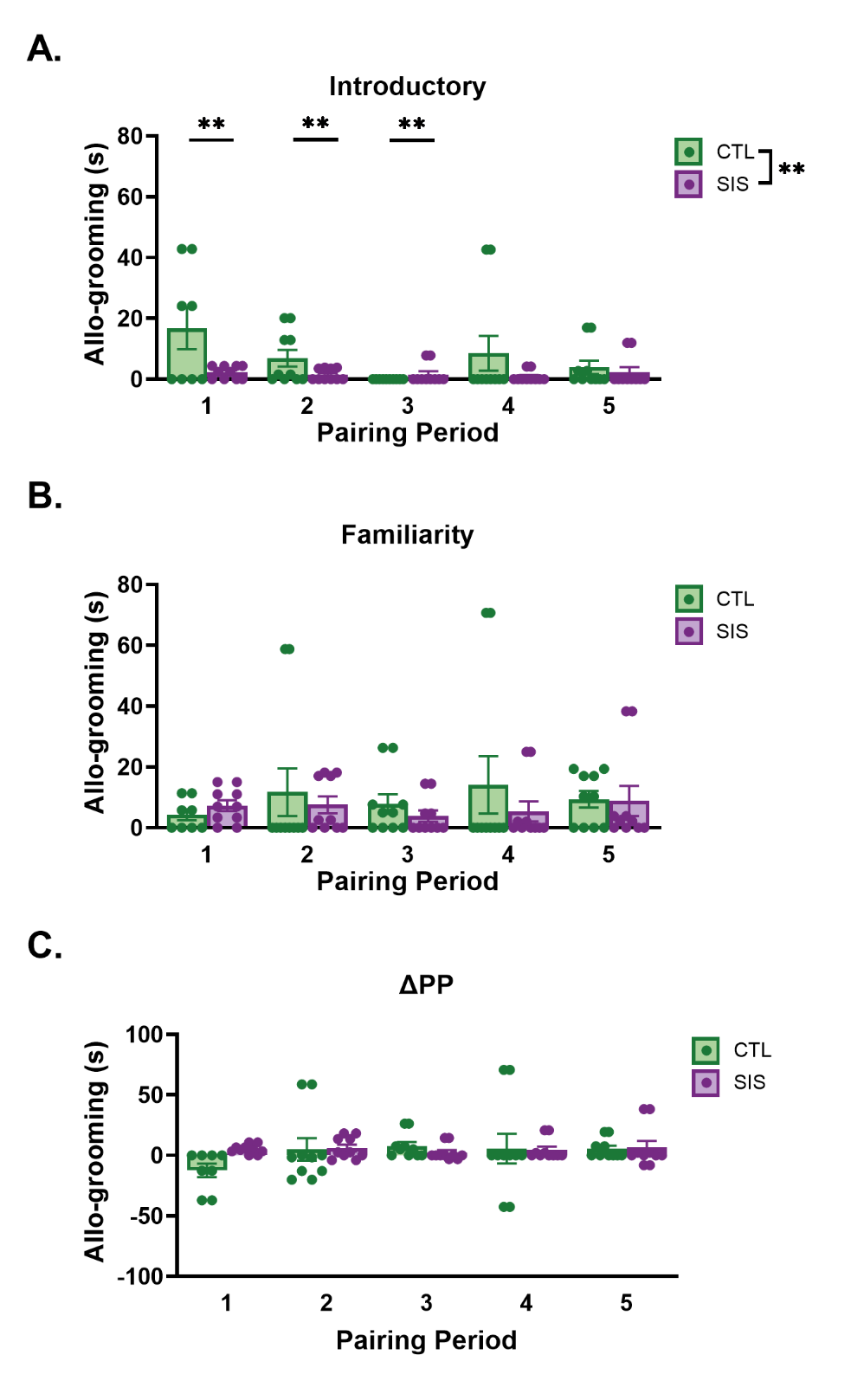


**Figure S1 | Duration of allo-grooming during home cage observation.** Introductory Periods 1-5 (A) occurred during postnatal days 31, 33, 35, 37, and 39, respectively. Familiarity Periods 1-5 (B) occurred during postnatal days 33, 35, 37, 39, and 41, respectively. (C) Change in all-grooming within Pairing Period (ΔPP_n_ = FP_n_ - IP_n_). Note that the y-axes differ between panels A-C. Data are presented as mean ± SEM and dots represent individual data (n = 8-10/group); CTL group shown in green dots and bars; SIS group shown in purple dots and bars; ***p* < 0.01.


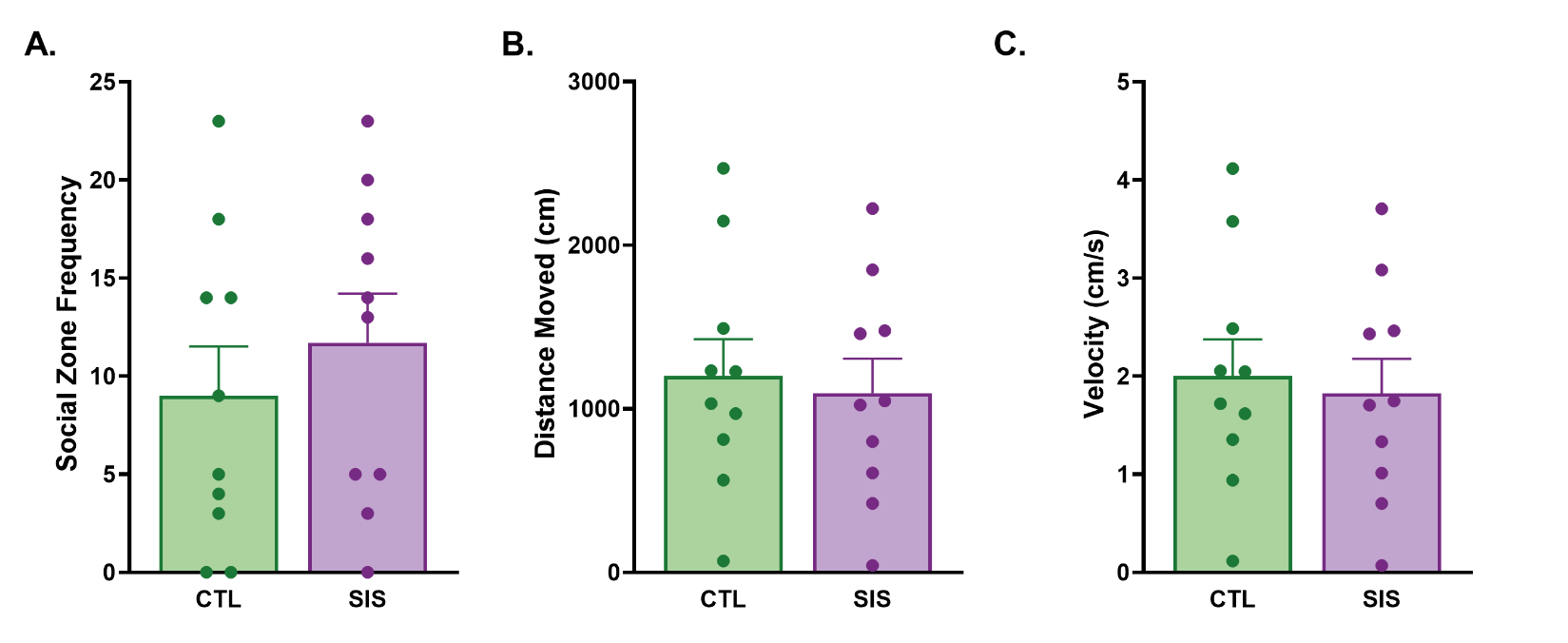


**Figure S2 | Social instability stress does not impact locomotion.** (A) Subjects in the CTL and SIS groups displayed similar frequencies to enter the social zone (A), distance moved (B), and velocity of movement (C) during the social approach test. Data are presented as mean ± SEM and dots represent individual data (n = 8-10/group); CTL group shown in green dots and bars; SIS group shown in purple dots and bars.

**
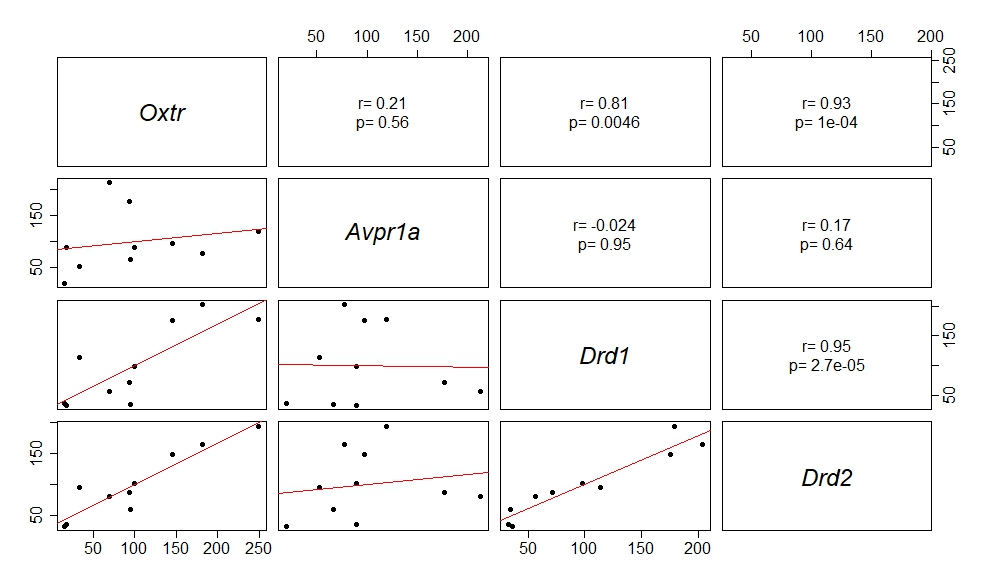
**

**Figure S3 | Correlation matrix between LS mRNA gene expression of the CTL group.** The upper triangular matrix shows the Pearson correlation coefficients (r) and corresponding *p*-values. The lower triangular matrix is composed of scatter plots with linear regression lines. The FDR adjusted alpha was α < 0.025 for the CTL group. Significant relationships correspond to Figure 9A.


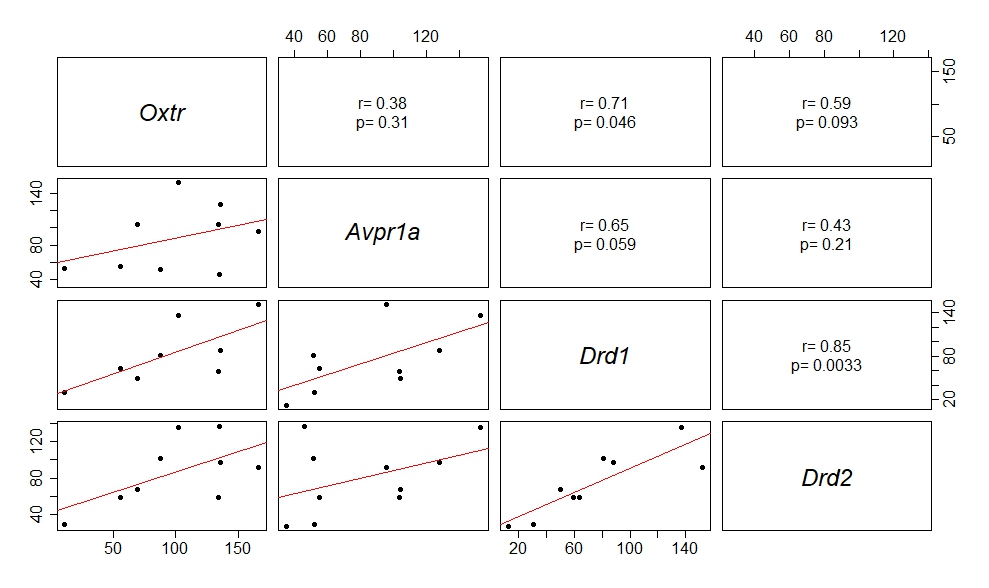


**Figure S4 | Correlation matrix between LS mRNA gene expression of the SIS group.** The upper triangular matrix shows the Pearson correlation coefficients (r) and corresponding *p*-values. The lower triangular matrix is composed of scatter plots with linear regression lines. The FDR adjusted alpha was α < 0.008 for the SIS group. Significant relationships correspond to Figure 9B.

**
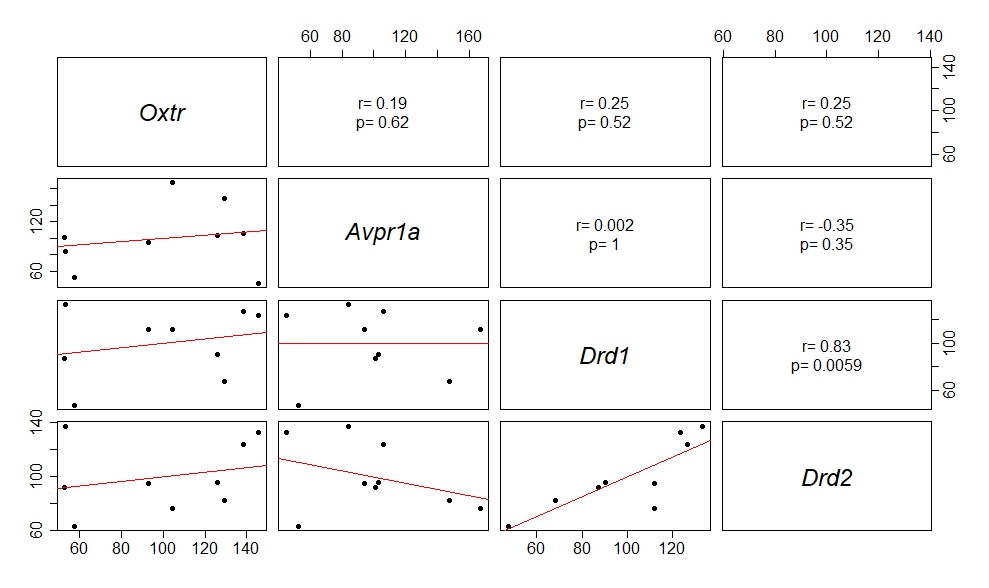
**

**Figure S5 | Correlation matrix between NAc mRNA gene expression of the CTL group.** The upper triangular matrix shows the Pearson correlation coefficients (r) and corresponding *p*-values. The lower triangular matrix is composed of scatter plots with linear regression lines. The FDR adjusted alpha was α < 0.008 for the CTL group. Significant relationships correspond to Figure 9C.


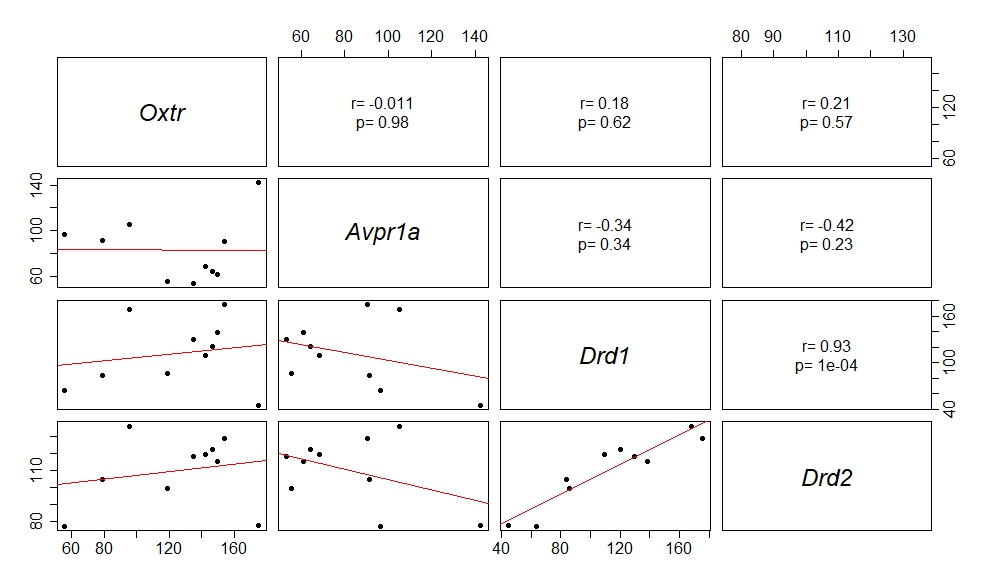


**Figure S6 | Correlation matrix between NAc mRNA gene expression of the SIS group.** The upper triangular matrix shows the Pearson correlation coefficients (r) and corresponding *p*-values. The lower triangular matrix is composed of scatter plots with linear regression lines. The FDR adjusted alpha was α < 0.008 for the SIS group. Significant relationships correspond to Figure 9D.

**
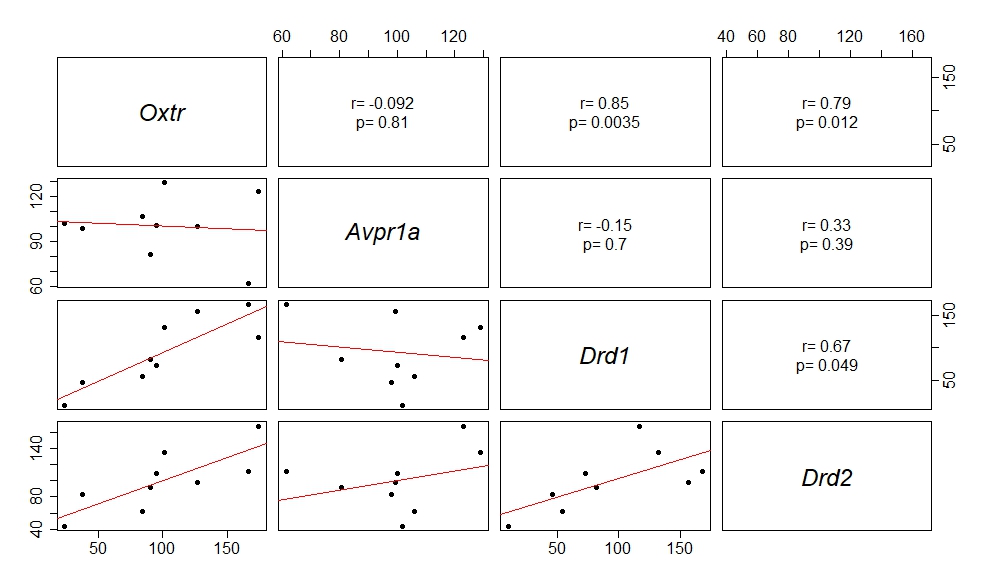
**

**Figure S7 | Correlation matrix between ACC mRNA gene expression of the CTL group.** The upper triangular matrix shows the Pearson correlation coefficients (r) and corresponding *p*-values. The lower triangular matrix is composed of scatter plots with linear regression lines. The FDR adjusted alpha was α < 0.017 for the CTL group. Significant relationships correspond to Figure 9E.


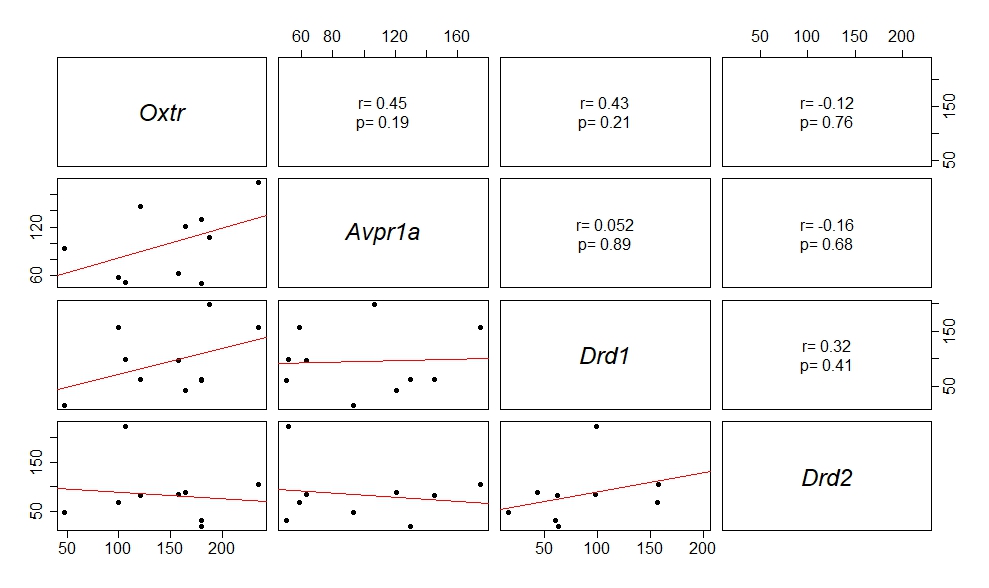


**Figure S8 | Correlation matrix between ACC mRNA gene expression of the SIS group.** The upper triangular matrix shows the Pearson correlation coefficients (r) and corresponding *p*-values. The lower triangular matrix is composed of scatter plots with linear regression lines. The FDR adjusted alpha was α < 0.008 for the SIS group. Significant relationships correspond to Figure 9F.
